# Neuromorphic Computing via Fission‐based Broadband Frequency Generation

**DOI:** 10.1002/advs.202303835

**Published:** 2023-10-02

**Authors:** Bennet Fischer, Mario Chemnitz, Yi Zhu, Nicolas Perron, Piotr Roztocki, Benjamin MacLellan, Luigi Di Lauro, A. Aadhi, Cristina Rimoldi, Tiago H. Falk, Roberto Morandotti

**Affiliations:** ^1^ Institut National de la Recherche Scientifique – Énergie Matériaux et Télécommunications 1650 Blvd. Lionel‐Boulet Varennes Quebec J3X1S2 Canada; ^2^ Leibniz Institute of Photonic Technology Albert‐Einstein Str. 9 07745 Jena Germany; ^3^ Ki3 Photonics Technologies 2547 Rue Sicard Montreal Quebec H1V 2Y8 Canada; ^4^ Dipartimento di Elettronica e Telecomunicazioni Politecnico di Torino Corso Duca degli Abruzzi 24 Torino 10129 Italy

**Keywords:** artificial neural networks, higher‐order soliton fissions, neuromorphic computing, nonlinear fiber optics, optics and photonics

## Abstract

The performance limitations of traditional computer architectures have led to the rise of brain‐inspired hardware, with optical solutions gaining popularity due to the energy efficiency, high speed, and scalability of linear operations. However, the use of optics to emulate the synaptic activity of neurons has remained a challenge since the integration of nonlinear nodes is power‐hungry and, thus, hard to scale. Neuromorphic wave computing offers a new paradigm for energy‐efficient information processing, building upon transient and passively nonlinear interactions between optical modes in a waveguide. Here, an implementation of this concept is presented using broadband frequency conversion by coherent higher‐order soliton fission in a single‐mode fiber. It is shown that phase encoding on femtosecond pulses at the input, alongside frequency selection and weighting at the system output, makes transient spectro‐temporal system states interpretable and allows for the energy‐efficient emulation of various digital neural networks. The experiments in a compact, fully fiber‐integrated setup substantiate an anticipated enhancement in computational performance with increasing system nonlinearity. The findings suggest that broadband frequency generation, accessible on‐chip and in‐fiber with off‐the‐shelf components, may challenge the traditional approach to node‐based brain‐inspired hardware design, ultimately leading to energy‐efficient, scalable, and dependable computing with minimal optical hardware requirements.

## Graphical abstract

This work presents an all‐fiber optical approach that harnesses the capabilities of nonlinear wave dynamics to process information. The study suggests that broadband frequency mixing in a single optical waveguide can mimic various digital neural networks, opening up a non‐intuitive use case for nonlinear photonics as a scalable resource for energy‐efficient brain‐inspired computing.

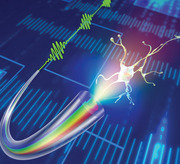

## 1 Introduction

The ongoing transformative success of artificial intelligence comes at the price of a significant environmental footprint.^[^ ^1^, ^2^ ^]^ The use of brain‐inspired algorithms on our current von Neumann computing architectures, which separate data processing and storage, requires a significant amount of extra energy to maintain continuous information exchange between units. A practical solution to this energy inefficiency is optical computing.^[^ ^3^, ^4^, ^5^ ^]^ Computing with light utilizes complex‐valued electromagnetic fields instead of electric currents to transport and process multi‐dimensional data. This enables parallel processing in various optical degrees of freedom^[^ ^6^ ^]^ at femtojoule energy levels per operation.^[^ ^7^ ^]^ Recent approaches aim to replicate mathematical core operations used in artificial neural networks (ANNs) – the current backbone of artificial intelligence – in ultrafast light‐driven hardware. Operations such as arbitrary matrix multiplications,^[^ ^8^ ^]^ convolutions,^[^ ^9^ ^]^ or nonlinear activation functions^[^ ^10^, ^11^, ^12^ ^]^ were implemented in multi‐component hardware to realize single optical neural network layers. Their inference capabilities are comparable to their digital counterparts in low‐level benchmark tasks, such as time series prediction or audio and image recognition.

Yet, further scaling of these design‐ and equipment‐heavy approaches toward deep neural architectures comes with many challenges. For instance, emulating the firing of a neuron necessitates programmable nonlinear optical interconnects which enact synaptic activation functions.^[^ ^13^, ^14^, ^15^ ^]^ These interconnects are power‐hungry and difficult to scale,^[^ ^16^ ^]^ making the sequential arrangement of optical neural nodes, unlike biological neurons, potentially impractical.

Neuromorphic wave computing (and related concepts^[^ ^17^, ^18^, ^19^ ^]^) may offer a solution to go beyond the limitations of conventional node‐by‐node hardware design. The analog computing principle relies on the natural wave dynamics of a physical system to perform computations rather than using complex heterogeneous hardware architectures with tailored information trajectories. Information is encoded in wave modes and processed in the complex‐number space via transient (temporary) wave phenomena, such as diffraction, interference, and nonlinear (i.e., intensity‐dependent) wave mixing. By training the timing of these phenomena, propagating waves may be able to resample transient nonlinear graphs comparable to nonequilibrium neural networks.^[^ ^20^, ^21^ ^]^ The concept is currently finding its way into hydrodynamics,^[^ ^22^ ^]^ acoustics,^[^ ^23^ ^]^ and optics.^[^ ^18^, ^24^, ^25^ ^]^

In the latter, wave computing comes with unique advantages. It presents an intrinsic, energy‐efficient approach to leveraging the ultrafast nonlinearity inherent to optical media. This not only promises to surpass the technological limitations in power consumption, data latency, and bandwidth of electro‐optical signal conversion – a major challenge in enabling deep (i.e., multi‐layer) all‐optical neuromorphic computing^[^ ^19^, ^25^ ^]^ – but may also allow for scaling the computational performance with the nonlinearity in the system.^[^ ^18^ ^]^

Yet, questions remain about the computational merit of using transient nonlinearities in various optical degrees of freedom, as well as which types of wave dynamics are sufficiently complex to perform scalable computations. Further, new metrics are needed to quantify the scalability of such analog systems in terms of neural performance and energy efficiency.

Here, we demonstrate the broadening of optical pulses in time and frequency within a single waveguide as a scalable resource for fast and powerful neuromorphic computing. Modes (i.e., elementary waves) in time and frequency provide an energy‐efficient vehicle to explore such an approach, as they are well‐understood and controllable in optical media, particularly in the context of broadband light generation.^[^ ^26^, ^27^ ^]^ We first revisit the analogy between neural networks and wave‐based neuromorphic computing. We then introduce coherent broadband frequency mixing mediated by the fission of higher‐order solitons (i.e., self‐regulating pulses) as a specific realization of such computing. The outstanding phase sensitivity of this complex phenomenon is ideal for nonlinearly transforming information encoded on a femtosecond data carrier into new frequency bands. Following this, we show how such frequency bands can be used for effortless data separation or prediction solely by training a linear mapping of the spectral intensities to a prediction label or value. Finally, we demonstrate this concept experimentally in an “off‐the‐shelf” fiber system and evaluate the system performance with various neural network benchmarks, including COVID‐19 diagnosis. We introduce network primitives, i.e., software‐based ANNs of minimal size with similar properties, as a new method to compare the performance of analog and digital platforms. Our results complement spatial approaches in nonlinear wave computing^[^ ^24^ ^]^ and earlier attempts to interpret narrowband frequency mixing in fibers as a computational kernel.^[^ ^28^ ^]^

## 2 Neuromorphic Wave Computing with Transient Nonlinear Optics

One way to understand why optical waves may compute in a neuromorphic manner is to visualize the analogous mathematical structure between deep neural networks and the differential equations that govern nonlinear wave propagation.^[^ ^17^, ^18^, ^25^ ^]^ This analogy is especially effective when utilizing time‐frequency modes for visualization (see Supporting Information Section A for a formal treatment).

**Figure 1:**
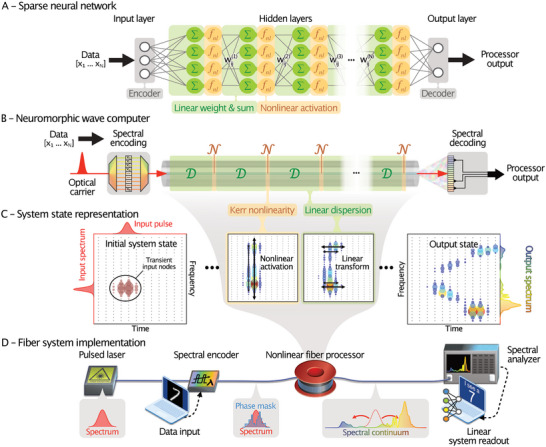
Operational principle of neuromorphic frequency‐domain wave computing. A) Example of a feed‐forward neural network architecture. Each hidden layer can be decomposed into a linear sublayer (green) performing node‐wise linear weighting and summation and a nonlinear activation layer (orange). B) Illustration of the heuristic resemblance between neural networks and fiber‐based neuromorphic wave computing. Information is encoded in the spectral phase of a femtosecond pulse before being launched into a highly nonlinear optical fiber. Nonlinear pulse propagation can be modeled as a concatenation of linear dispersive shifts D and third‐order optical nonlinear transformations N (i.e., the Kerr effect), leading to the mixing and generation of optical frequencies. The broadband system output is then measured and weighted to provide a task‐specific computational result. C) System state representation illustrating the information flow in a transient optical network while propagating in the spectro‐temporal space. An input field is represented as a finite distribution of weights on specific transient nodes, i.e., transform‐limited field entities in time and frequency. Continuously alternating linear dispersion and nonlinearity will cause energy redistribution along time and frequency, respectively, creating highly input‐specific information trajectories. In the visualization, the field amplitudes per node are coded in both the size and color of the shape for better visibility. D) Schematic setup of our experimental realization (see Figure S1, Supporting Information for more details). The system is trained offline by frequency selection and weighting at the system read‐out.

In an artificial neural network, such as that depicted in **Figure** **1**A, each neural layer typically comprises several interconnected neurons. Each artificial neural node in layer *k* can mathematically be expressed as^[^ ^29^ ^]]^

(1) $$X_{p}^{k}=\;f_{NL}\left(\sum_{q}w_{pq}^{k}X_{q}^{k-1}\right)$$

Each neuron accumulates weighted information $X_{q}^{k-1}$ from previous nodes, followed by a nonlinear activation function *f_NL_* ( · ) (usually given by sigmoids or rectifying units). Such a nonlinear activation resembles the synaptic response of a biological neuron while the weights $w_{pq}^{k}$ of each layer mimic the plasticity of biological neural connections. The activation threshold and weights are usually subject to task‐specific training. The interconnections may be random and sparse as long as the network complexity is sufficient to address the desired inference tasks.^[^ ^21^, ^30^ ^]^ In general, a multi‐layer network can be interpreted as a sequence of linear and nonlinear operations (Figure 1A).

This sequence is similar in formalism to the split‐step Fourier model, which is commonly used to simulate approximate nonlinear pulse propagation in optical waveguides.^[^ ^26^ ^]^ This model applies linear dispersion and nonlinear wave mixing repeatedly and consecutively in a dense sequence of operations (Figure 1B). A single propagation step *k* can formally be expressed, analogously to Eq. 1, as:

(2) $$A_{p}^{k}=\;N\left(\sum_{q}D_{pq}A_{q}^{k-1}\right)$$

The weighted sum corresponds to the linear temporal response of the physical system $D_{pq}$ applied to the complex‐valued temporal field amplitude$A_{p}$. The sum can be understood as a discretized convolution of the field with the dispersive system response, delayed by a time *T_p_* − *T_q_*. Finally, the nonlinear operator N, in our case the third‐order Kerr nonlinearity, acts as synaptic activation.

The information flow in an optical waveguide can be visualized as a virtual neural network of transient nodes, as depicted in Figure 1C. Such nodes are aligned in a two‐dimensional space in time and frequency. An optical field input into the network corresponds to complex‐valued nodes at a specific time and frequency. At every tiny step of the wave propagation, the linear dispersion D causes the information to spread out over time (Figure 1C, green box), while the nonlinearity N mixes either the nodes overlapping in time or maps their product into other frequencies (Figure 1C, orange box). Hence, the interplay between linear dispersion and nonlinearity mimics a transient flow of information through a neural network. The system's wave dynamics define the connections within this network, which are unique for a given system configuration (i.e., pulse and waveguide properties).

We stress that these heuristic assumptions, while intuitive, cannot be used to deduce quantitative rules to reconstruct conclusive graph topologies of the neural networks that the system can mimic. Furthermore, they cannot be used to estimate the computational costs, such as the number of operations performed per time.

To conclude the analogy to neural networks, trainable parameters are required to direct and blend information toward a prediction value. In ANN algorithms, these parameters typically refer to the weights of the neural interconnections between layers. In wave systems, the free parameters are(a) the initial configuration of the input field; b) the system nonlinearity and dispersion (i.e., both define the dominant nonlinear dynamics of the system); and c) the field parameters read from the system output. Thus, a physical wave system may be trained toward specific inference tasks through a priori design of the waveguide for tailored wave propagation,^[^ ^17^, ^19^, ^25^ ^]^ or a posteriori by optimizing the system encoding (input layer) or read‐out (output layer). In practice, the training of the waveguide parameters is very challenging as it requires precise control over local waveguide properties, such as dispersion and nonlinearity, which are typically static after fabrication. While some solutions to this challenge exist (e.g., dispersion‐varying fibers^[^ ^31^, ^32^, ^33^ ^]^), a posteriori training at system input or output is more straightforward toward tailoring the nonlinear wave dynamics. This approach exploits the system's sensitivity to phase and amplitude variations at the input. As a result, task‐specific transient information graphs in nonlinear waveguides can be trained in two ways: either online, by adjusting a phase/amplitude mask of the input field^[^ ^34^ ^]^ (known as input layer training), or offline, by weighting the system read‐out. The latter is known as output layer training from computational concepts such as reservoir computing^[^ ^35^, ^36^ ^]^ or extreme learning machines (ELM).^[^ ^18^, ^30^ ^]^

Overall, a general prerequisite for wave computing is a physical system that: i) features a sufficiently complex, input‐dependent system response (i.e., a manifold of modal interactions); ii) remains robust against noise; and iii) yields interpretable, reproducible outputs.^[^ ^21^ ^]^ Processes such as the formation of self‐regulating states of light^[^ ^18^, ^38^ ^]^ (e.g., solitons) or the scattering of spatial modes^[^ ^17^ ^]^ have been theoretically deemed suitable for computing, and nonlinear spatial mode interactions in optical fibers have been demonstrated experimentally.^[^ ^24^ ^]^ Spatial modes dominate in current demonstrations. They are easily scalable yet require high power and environmental stability. In the following, we introduce fission‐based broadband frequency generation – a complex interaction of robust time‐frequency modes – as a novel, versatile basis for neuromorphic wave computing in conjunction with read‐out training.

## 3 Results

### 3.1 Higher‐Order Soliton Fission For Neuromorphic Wave Computing

Soliton fission is a multi‐step nonlinear process leading to the generation of new frequencies over a bandwidth of several tens of terahertz.^[^ ^37^ ^]^ The process features one of the most complex yet coherent and tailorable pulse dynamics known in optics. It involves the sequential split‐up of pulses into a series of optical solitons, often accompanied by a cascade of other nonlinear effects such as self‐phase modulation, non‐solitonic radiation (i.e., dispersive waves), intrapulse stimulated Raman scattering, and four‐wave mixing, to name a few. Recent studies have demonstrated that fully connected deep neural networks with at least two hidden layers are required to sample the full field dynamics of the soliton fission process,^[^ ^38^ ^]^ unambiguously supporting the non‐trivial input‐output relationship of such a physical system.

**Figure 2:**
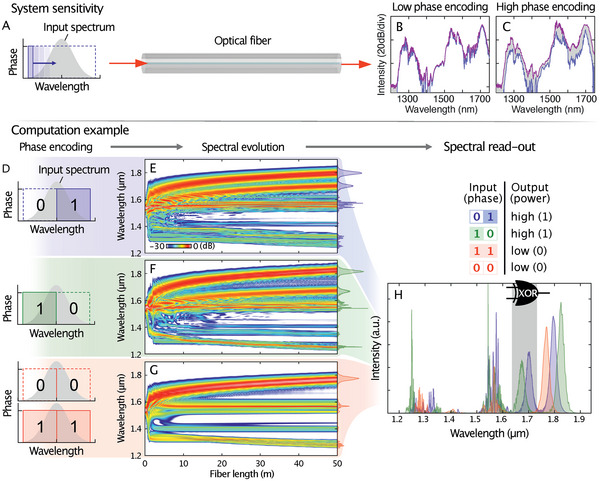
Coherent soliton fission as a computing resource. A–C) System sensitivity analysis. A) A single‐phase window (called *bit*; in blue) is shifted through the spectrum of a pulse input to a nonlinear fiber (in gray). B,C) Output spectra of a 100 m long nonlinear fiber measured for 72 different spectral bit‐positions. The variations in the spectrum demonstrate the phase sensitivity of the nonlinear broadening process for B) low phase magnitude (π/10) and C) high phase magnitude (π) (see also Figure S3, Supporting Information). The purple and blue lines indicate the maximum and minimum values per spectral read‐out (called *bin*), respectively. D) Schematics for encoding the nonlinear 2‐bit parity (XOR) problem in the spectral phase of a femtosecond pulse. E–G) Simulated spectral evolutions of the femtosecond pulse over 50 m of commercial highly nonlinear fiber for each input encoding in (D). H) Intensity spectra measured at the fiber output for each input in (D). The unique information trajectories in (E–G) yield easily separable intensity features in the output spectra. This allows the XOR problem to be solved by measuring the power in a single wavelength band (see table inset) indicated by the grey area in (H).

The timing and strength of each component in this dynamic cascade of effects strongly depend on the configuration of the optical input pulse. The slightest alteration in phase or amplitude of the incident pulse spectrum can lead to a significant change in the output spectrum in a coherent and reproducible way, as shown in **Figure** **2**A–C (see also Supporting Information, Section B and Figure S3, Supporting Information). The rich variety of nonlinear transformations in such complex, cascaded wave dynamics enables the basic mapping functionalities of various neural networks to be emulated with a single physical unit.

**Figure 3:**
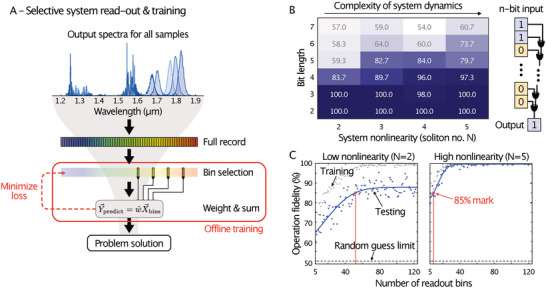
System training and solving the n‐bit parity problem. A) Flowchart of the digital processing layers to interpret the system readout. The training is performed offline using bin selection and linear regression. A simple search algorithm iterates through different frequency bin combinations (see Experimental Section). For each combination, linear regression is used to predict the label (or value) of an inference task. The prediction error was estimated through cross‐validation of subsets of the training data. The best‐performing combination of bins (i.e., lowest loss) defines an inference‐ready system configuration. B) Experimentally measured operation fidelity associated with the n‐bit parity problem for increasing bit length and system nonlinearity. The latter is given in units of soliton number *N* (Experimental Section). The best performance is achieved at higher system nonlinearity. C) Experimentally measured operation fidelity for a 5‐bit parity problem versus increasing the number of readout bins for low (left panel) and high (right panel) system nonlinearity. Higher system nonlinearity requires fewer readout bins for optimal performance since a higher degree of frequency mixing leads to a larger set of possible data projections. For instance, 52 bins are required at low nonlinearity to achieve 85% inference accuracy (see red line in C), while only 10 bins are needed at high nonlinearity.

We illustrate numerically (Figure 2D–H) and demonstrate experimentally (**Figure** **3**B,C) the potential of soliton fission as the backbone for neuromorphic wave computing by means of the nonlinear exclusive OR (XOR) operation (Experimental Section and Supporting Information). In our approach, each combination of a 2‐bit sequence is encoded in the spectral phase of the input pulse (Figure 2A,D). The simulation results (Figure 2E–G) show a significant difference in the nonlinear system dynamics between input sequences with odd ([0,1], [1,0]) and even ([0,0], [1,1]) parities. Consequently, distinct broadband areas can be identified in the output spectrum (e.g., at 1700 ± 50 nm – Figure 2H) that yield high power for odd‐numbered inputs (an XOR result of 1) and low power for even‐numbered inputs (an XOR result of 0). This example highlights both the sensitivity of the soliton fission process to the input phase (Figure S3, Supporting Information) and the system's intrinsic ability to perform digital operations beyond the capabilities of a single perceptron.^[^ ^39^ ^]^

### 3.2 Experimental Implementation and Benchmarking

We experimentally implemented a nonlinear fiber system (Figure 1D) consisting of a femtosecond laser source, a programmable filter for data encoding, and a highly nonlinear, anomalous dispersive fiber for data processing (see also Experimental Section and Figure S1, Supporting Information). The system is dispersion‐optimized to obtain an input pulse width (≈140 fs) at the input facet of the nonlinear fiber in order to maintain pulse‐to‐pulse reproducibility of the transient soliton fission dynamics (also known as coherence condition, see Experimental Section and Figure S2, Supporting Information). This optimization can be understood as adding a bias to the neuromorphic system that provides another means for managing the system on top of the regulation with optical input power.

The fiber processor is trained toward individual operations and inference tasks using the ELM framework (i.e., offline, supervised readout layer training).^[^ ^30^ ^]^ The trainable parameters are the selected number of measured frequencies and their weights. In contrast to other studies, we strictly omit smart nonlinear layers, such as convolutional neural networks or (nonlinear) support vector machines, which either preserve data dimensionality or use the entire output information of the physical layer as an input for digital classifiers. Instead, we use linear regression on only a selected set of measured spectral intensities to obtain a weight matrix from the system readout (Figure 3A). The selection of frequencies is a further degree of freedom in our system training that allows us to learn about the complexity of an inference task (i.e., how many inputs are needed for each specific nonlinear regime) and the performance scaling of the proposed approach.

With this setup, we confirm the significant advantage of physical ELMs: the computational performance of our system can be scaled up without changes to the optical hardware simply by increasing the complexity of the underlying nonlinear dynamics. The extent and reach of nodal interactions in the transient space depend on the amount of nonlinear optical processes involved in the dynamics, the complexity of which is likely to affect the maximal network topology that a wave computer can mimic. Nonlinear dynamics that undergo multiple stages and interactions, such as higher‐order soliton fission,^[^ ^26^, ^37^ ^]^ may even allow us to reproduce the performance of multi‐layer or deep neural networks with relatively low training.^[^ ^19^, ^25^ ^]^

In Figure 3, panels G and H, we experimentally show this intrinsic system scalability (the training results are presented in Figure S5, Supporting Information) for the generalized XOR operation (Experimental Section). For a given nonlinearity, as defined by the soliton number *N* (Experimental Section), misclassification becomes larger for increasing bit‐lengths. We note that rising the system nonlinearity *N* (e.g., by means of higher optical power) improves the operation's fidelity and computational performance per given bit length. We observe that in the case of high nonlinearity (*N* = 5), the discrepancy between training and test accuracy is lower compared to the case of low system nonlinearity (*N* = 2).

This observation indicates that the system's aptitude for generalization (i.e., capability to infer from unseen data) improves when the complexity of its dynamics is higher. A similar behavior has been reported for ANN algorithms for increasing network size.^[^ ^40^ ^]^ Richer dynamics lead to a larger variety of frequency mixing and conversion processes, effectively increasing the dimension of the feature space at the output. A simple search algorithm based on equally spaced frequency bins, as we use in this work, has, hence, a much higher chance of finding a combination of data projections that fit an expectation value more accurately than in a system featuring low nonlinearity. Increasing the number of readout frequencies has a similar effect in improving prediction accuracy, as shown in Figure 3C (see Figure S6, Supporting Information for another example).

**Figure 4:**
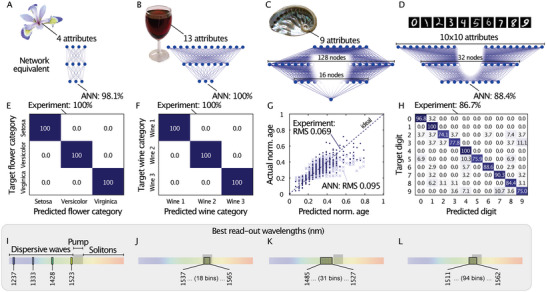
Task‐dependent benchmarks commonly used to evaluate neural networks. A–D) Digital artificial neural network primitives for the A) *IRIS*, B) *WINE*, C) *Abalone*, and D) *MNIST* tasks, featuring equal performance as our experimental fiber‐based processor. E–H) Operation accuracy of experimentally obtained data for the encoded test sets. The system was operated under the same conditions for all sets. I–L) Spectral position (in nanometers) of the readout bins that yield the best operational performance at 0.5 nm bin width.

Broadband frequency conversion is capable of addressing a fundamental problem in neuromorphic computing, namely solving entirely different classes of tasks (e.g., universal nonlinear regression (Experimental Section and Figure S4, Supporting Information), categorical regression, low‐ and high‐feature‐number classification) without redesigning the computing basis, i.e., the underlying neural network architecture. We assess our system's neuromorphic performance and flexibility by experimentally performing several task‐dependent, standard benchmarks with varying feature complexities. The benchmarks tested included orchid flower classification (*IRIS* dataset), wine classification (*WINE* dataset), age prediction of sea snails (*Abalone* dataset), and image recognition of handwritten digits (*MNIST* dataset); see Experimental Section for details. The inference results are illustrated in **Figure** **4**E–H (see Supporting Information, in particular, Figures S7,S8,S10, Supporting Information for training results). The hyper‐parameters used for each set are summarized in Table S1 (Supporting Information). The input features were normalized to the same maximal phase shift (i.e., π⁄8) before being encoded in the spectral phase across the C‐band (Experimental Section and Figure S2, Supporting Information). The maximal phase shift was chosen to achieve a higher variation in the spectral system output with minimum disturbance to the input pulse shape (Figure S2, Supporting Information).

For the IRIS and WINE tasks, the system achieves a classification precision of 100% for unseen test data, outperforming other optical approaches (see Table S2, Supporting Information). The Abalone task is a regression task based on 13 input categories, which our system can solve effortlessly with a root‐mean‐square (RMS) error <0.07. In the MNIST task, however, our system achieves only moderate accuracies of 86.7% in assigning 10‐digit labels to handscript images. In experiments with smaller sample batches (here 300 random images), we found that the prediction accuracy generally improves with a higher number and smaller spectral bandwidth of read‐out bins. Yet, results always remained below 90%. We hypothesize that the strong nonlinear mixing of information by our system could be detrimental in the case of MNIST, as the dataset may require significantly less nonlinearity to become separable. Other demonstrations have achieved higher accuracies (>90%) with purely linear digital^[^ ^41^ ^]^ or mildly nonlinear optical^[^ ^42^, ^43^ ^]^ networks.

**Figure 5:**
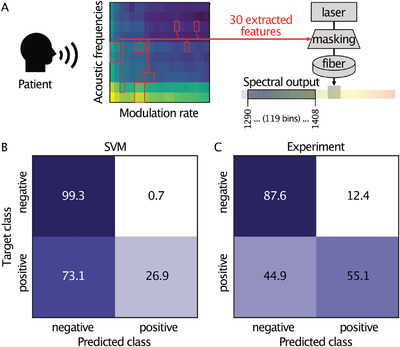
COVID‐19 diagnosis from digital speech audio recordings. A) Simplified scheme for feature extraction based on principle component analysis of processed audio signals. The same selected features have been used as input to both digital and optical classifiers for direct comparison. B) The test results for the digital support vector machine (UAR = 0.631) and C) The test results of the experimental fiber‐based ELM implementation (UAR = 0.7133) show a notable performance increase of our approach compared to a digital state‐of‐the‐art technique.

A particular advantage of the proposed ultra‐short pulse‐based optical schemes is a low and constant energy usage per information carrier, largely independent of the chosen task. For instance, for all results in Figures 4 and **5**, our system was operated under identical conditions (89 pJ pulse energy, 140 fs pulse duration, a soliton number of 5, see also Supporting Information, Section G). To evaluate the potential computational advantage of our technique, we identified network primitives for each task, i.e., minimal‐sized software‐based ANNs with performance benchmarks similar to our experimental system. As shown in Figure 4, A to D (see Table S3, Supporting Information for hyper‐parameters), they require vastly different efforts in training and energy consumption as opposed to the constant configuration of the fiber‐based ELM approach (Table S4, Supporting Information), highlighting the energy efficiency and ease‐of‐use of this scheme. We also note that even with dedicated efforts to alter the structure and node activations of our ANN configurations, none performed better than our experimental system in the Abalone task.

Finally, we have also tested our system in a real‐world scenario, using the diagnostics of COVID‐19 patients from audio samples (INTERSPEECH 2021 challenge^[^ ^44^ ^]^). Based on 30 extracted features of each audio sample, we obtained a competitive prediction accuracy of 77.1%, which is higher than the best software classifier we could identify for unbiased data (state‐of‐the‐art support vector machine with 72.9% accuracy; see Figure 5, Figure S10, Supporting Information, and Experimental Section).

## 4 Conclusion

Broadband frequency conversion in optical fibers can perform various tasks commonly undertaken by artificial neural networks at just a fraction of their training complexity and energy consumption per inference. The results support the use of transient system dynamics,^[^ ^21^ ^]^ particularly optical nonlinear dynamics, to emulate the functionality of multiple neural network topologies. To ensure trainability and reproducibility, coherence (i.e., pulse‐to‐pulse spectral stability) must be maintained at all times. In anomalous dispersive fibers, this can only be achieved by strictly adhering to the outlined guidelines^[^ ^26^ ^]^ which includes keeping the soliton number *N* below 10 and pulse durations well below 200 fs. The latter requires advanced dispersion compensation techniques in fiber‐integrated systems and low‐phase altitudes for data encoding.

Our accessible implementation uses exclusively off‐the‐shelf telecom fiber components and is, in principle, transferable to on‐chip nanophotonic devices^[^ ^45^, ^46^ ^]^ and novel material fiber systems.^[^ ^33^, ^47^ ^]^ In particular, photonic chip technologies offer full system integration, higher energy efficiency, and potentially picosecond inference latencies for cm‐scale waveguides. Also, on‐chip solutions offer highly reproducible waveguide properties and might allow for the direct transfer of trained weights from one chip to another. Yet, novel fiber systems are widely accessible and can open new research grounds to further explore uncommon propagation dynamics for neuromorphic wave computing. For example, specially designed dispersion with more than one or varying zero‐dispersion wavelengths might enrich the cascade of nonlinear effects by multiple, locally distributed dispersive emissions and four‐wave mixing events.^[^ ^31^ ^]^ In addition, current developments of highly nonlinear non‐silica fibers^[^ ^47^, ^48^ ^]^ or dispersion‐engineered fibers^[^ ^49^ ^]^ might allow for a further reduction in the energy consumption to sub‐pJ per computation.

In general, each nonlinear optical process sensitive to phase and amplitude modulations can be used for such information processing. Yet, the achievable performance may differ with the dynamic range of the respective system output (e.g., bandwidth and spectral sensitivity to the input field may vary). Overall, frequency‐domain approaches involving second‐order^[^ ^13^, ^14^, ^50^ ^]^ or third‐order nonlinearity (this work) seem to be a particularly promising degree of freedom regarding sub‐pJ energy consumption per inference. Nonlinear systems that operate on the Kerr effect (e.g., soliton fission, four‐wave mixing) would come with the additional benefit of frequency windows that are widely customizable for a wide range of optical amplifiers and cascaded operations.

Our experiments have shown evidence for improving computational performance with increasing system nonlinearity, confirming earlier theoretical predictions in non‐dissipative multi‐soliton systems.^[^ ^18^ ^]^ In summary, these findings indicate a new approach to designing neuromorphic hardware. Instead of building neural hardware one node at a time, the system's inherent dynamics can be used to scale and enhance its inference capabilities. Yet, in all anomalous dispersive systems, the coherence conditions (i.e., *N* < 10) impose an upper bound to the scalability of the system's computational performance.

Moreover, by closely tracking our system accuracy with equally performing neural network primitives, we find that our fiber‐based processor can emulate a variety of neural networks, including multi‐layer networks (see Abalone results), with a single system setting. Considering single pulse inference and waveguides with tailored dispersion, it is possible to attain energies as low as a few pico‐joules per inference, regardless of the task at hand. This outperforms current GPU performance by two to three orders of magnitude (see Supporting Information, Section G), which contradicts some recent assumptions that nonlinear optics lacks energy efficiency for computing.^[^ ^51^ ^]^ However, while pulse‐wise spectral measurements exist (e.g., time‐stretch techniques^[^ ^52^ ^]^), new approaches to scalable ultrafast electronic‐to‐optical interfaces are required to enable information encoding at such bandwidths and rates.

Overall, computing with transient nonlinear dynamics may open opportunities in developing a new generation of versatile, cost‐ and energy‐effective neuromorphic hardware for future sustainable photonic computing and machine learning applications.

## 5 Methods

### Experimental System Implementation

The setup for the experimental realization of our transient optical neural emulator is illustrated in Figure S1 (Supporting Information). It comprises off‐the‐shelf, polarization‐maintaining (PM), fiber‐coupled components and devices, thus ensuring turn‐key stable operation with a reasonably compact footprint. A repetition‐rate stabilized femtosecond laser (Menlo FC1500‐250‐WG; 250 MHz repetition rate) with ≈70 nm bandwidth centered at ≈1556 nm was used as a broadband optical source. The laser emits pulses that were stretched to >2 ps. The laser output was fed into a dispersion‐compensated erbium‐doped fiber amplifier (Pritel PM‐SPFA‐23) to compensate for subsequent device and coupling losses. A customized, fully polarization‐maintaining, programmable spectral filter (Finisar Waveshaper 1000A/X, slow axis working only) was used to apply dispersion compensation and imprint phase information onto the pulse spectrum (C‐band: 1528–1568 nm; see also Figure S2, Supporting Information). Furthermore, a dispersion‐compensating fiber (Thorlabs PMDCF, dispersion = $-100\;\frac{\mathrm{ps}}{\left(\mathrm{nm}\cdot \mathrm{km}\right)}$, length ≈4.5 m) was used to pre‐compress the dispersed optical pulses (i.e., >2 ps) down to ≈400 fs (see Figure S2A, Supporting Information) before entering the highly nonlinear fiber (OFS Fitel, HNLF‐PM, dispersion = $1.416\;\frac{\mathrm{ps}}{\left(\mathrm{nm}\cdot \mathrm{km}\right)}$, nonlinear coefficient = 10.7 W^−1^km^−1^, length = 100 m). The output was measured with an optical spectral analyzer (ANDO AQ6317B).

It was noted that the amplification causes a nonlinear broadening, which cannot be fully compensated with the Waveshaper. Nonetheless, the additional nonlinear phase remains constant, and pulses were identical prior to the phase encoding of data.

### Autonomous Pulse Optimization

Femtosecond pulses below 200 fs duration at the input of the highly nonlinear fiber (HNLF) were a prerequisite for pulse‐wise reproducibility of the broadband output spectra. The dispersion compensating fiber (DCF) used in these experiments can pre‐compress the pulses down to ≈375 fs (see Figure S2A, Supporting Information), as measured before they reach the HNLF (pigtail to HNLF taken into account). A residual dispersion was caused by fabrication tolerances of different fiber types (i.e., featuring slightly different dispersion coefficients) and limited control over fiber length (i.e., a mismatch of 10 cm lengths was possible). Thus, additional pulse compensation was required and was achieved by adding a custom phase mask on the programmable spectral filter. This phase mask was based on a 5^th^‐order polynomial, the coefficients of which were found using a particle swarm optimization algorithm to reach the shortest pulse duration, similar to earlier approaches with genetic algorithms.^[^ ^53^ ^]^

The polynomial phase profile was formally expressed as:

(3) $$p\left(\nu \right)\;=\;\sum_{n\;=\;0}^{5}\pi^{n}\left(n+1\right)!\cdot q_{n\;}{\left(\nu -\nu_{0}\right)}^{n}\cdot 10^{-2\left(n-1\right)}$$

with frequency range ν and center frequency ν_0_ of the Waveshaper, and coefficients *q_n_* which were subject to the system optimization. The last factor was an empirically found scaling factor to adjust the impact of higher‐order dispersion terms. The coefficients found through the system optimization were *q_n_* ϵ [0.0367, 0.4551, 0.6852, 0.9940, 0.1275, 0.5120], while the first entry corresponds to *n = 0*. Yet, it was noted that these coefficients depend on the system configuration, such as laser source, incorporated fibers, and their respective lengths, etc., and were generally unique to a particular fiber system. Therefore, the optimization must be repeated for different systems. Nonetheless, for a comparable system implementation (i.e., similar pulse parameters and overall fiber system dispersion) the found coefficients could be used as initial starting parameters to accelerate the convergence of the optimization algorithm.

A commercial autocorrelator (Femtochrome FR‐103XL) was used for pulse duration monitoring during the optimization phase. A two‐term Gaussian function was fitted to the measured autocorrelation for background evaluation in relation to the main feature. The results of the pulse optimization are shown in Figure S2A,B (Supporting Information) where the 375 fs pulse was compressed to ≈ 140 fs (full width at half maximum) before the HNLF.

### Data Encoding

Information in our system was encoded in the spectral phase rather than the optical amplitude of the optical pulse to maintain high pump energies (11). Information inputs *M* (i.e., given by the features $f_{i}^{M}$ of sample *i* in a K‐sized data set) was normalized per feature group, i.e., $f_{i}^{M}=f_{i}^{M}\;/max\left(f_{1}^{M},\;f_{2}^{M},\;\text{…},\;f_{K}^{M}\right),$ and converted into phase values by multiplying the feature values with a constant phase factor. This factor was determined by the iterative optimization described below. The feature mask was added to the custom phase mask required for dispersion compensation. Finally, the composite phase mask was imprinted onto the pulse spectrum using the programmable filter.

This filter offers up to 400 individual bins across a bandwidth of 72 nm (i.e., 1528–1600 nm). Each bin allows for imprinting phase values between 0 and 2π. Encoding was limited to the C‐band (1528–1568 nm) for all data sets, while the attenuation was chosen to be zero over the entire C‐ and L‐bands. The broadband transmission of the filter was crucial to achieving pulse durations below 150 fs, given that it was impossible to maintain coherent soliton fission as the dominant broadening effect for longer pulses.^[^ ^26^, ^37^ ^]^ An encoding example is shown in Figure S2C (Supporting Information).

### Data Acquisition

The optical spectral analyzer was interfaced with a computer, recording the data in mW (linear scale) from the spectrometer to avoid superimposing nonlinear transformations besides the unavoidable photodiode signal acquisition. It was noted that, in principle, it was possible to achieve a more straightforward configuration by replacing the spectrometer with spectral filters and photodiode arrays. Any further normalization of the recorded data, such as softmax(⋅), commonly used by the neural network community, was not considered.^[^ ^29^ ^]^ To account for the trade‐off between resolution and acquisition time, a resolution ranging from 0.2 nm to 0.55 nm was chosen, together with 1000 to 2000 sampling points (depending on the task) to cover the entire 550 nm bandwidth of the spectrometer (i.e., 1200–1750 nm). The dependency on the spectral resolution has been studied in detail for the MNIST benchmark (see Figure S8, Supporting Information).

### Training

The underlying training framework for this system implementation resembles an extreme learning machine (ELM).^[^ ^30^ ^]^ The ELM concept relies on randomly connected hidden‐layer nodes in a feed‐forward configuration. Such a system is trained exclusively at the output layer using linear regression.

To find the best spectral readout bins, an equal search algorithm was utilized to determine well‐performing wavelength combinations: For a chosen number of frequency channels (i.e., bins), the spectral location of the first bin and the distance between all readout bins were iteratively varied. Each resulting set of frequencies was used for training and evaluation. The training was based on a *p*‐fold cross‐validation method using the mean square error (MSE) as a loss function.^[^ ^54^ ^]^ For cross‐validation, the training set was split into *p* numbers of subsets. *p*‐1 sets were used for linear regression, and the remaining set to estimate the loss value (MSE). This process was repeated for all circulating permutations of the *p* subsets. The best MSE of all permutations was recorded for a selected number of readout bins, determined according to the task. Subsequently, the bin combination and corresponding weight matrix yielding the lowest MSE for testing were selected. The equal search approach requires a few seconds to minutes to determine the best read‐out frequency channels and their weights, depending on the chosen amount of readout bins, the number of samples, and the resolution of the spectral recordings. For most tasks presented here, a 5‐fold cross‐validation method was used (see Table S1, Supporting Information).

For classification, the system was trained toward one‐hot output labels (sometimes referred to as “1‐of‐K coding”^[^ ^29^ ^]^). Here, the target output vector *Y_Target_* was an array of *K* entries representing *K*‐number classes, with the target class entry being 1 and all other entries being 0. To retrieve a singular prediction from the measured output vector *Y_pred_*, which was generally non‐binary, the “winner‐takes‐it‐all” approach was applied as decision boundary (i.e., the argmax*(Y_pred_)* operation). This returned the index of the maximum value in *Y_pred_*, thus defining the winning class.

Finally, a trained weight matrix was applied to the selected (best) readout bins of unseen test data to perform prediction tasks, as shown in Figures 4 and 5 of the main text. The corresponding training times for the neuromorphic processor are listed in **Table** **1**. Note that the summarized training times correspond only to the digital part of our training method (bin search, cross‐validation, and linear regression) and do not include the experimental acquisition times (see Supporting Information, Section C).

**Table 1:** Overview of offline training times for each task of the neuromorphic processor.

Task	IRIS	WINE	ABALONE	MNIST	COVID
No. of read‐out bins	4	18	31	94	119
Total training time (s)	68.2936	9.1001	23.4792	3.6329	6.839745

### Data Sets

All data sets for the task‐dependent benchmarks were publicly available (see Data Availability Statement and Figures S4,S5,S7,S10, Supporting Information). Below, the task‐independent benchmarks (i.e., n‐bit parity and nonlinear function regression) and the pre‐processing of the MNIST and COVID‐19 data are described.

*n‐bit parity* – The generalized XOR operation (n‐bit parity) acts on a vector $\vec{B}$ containing *n* uniformly distributed pseudo‐random integers *b_k_* from the interval [0, 1]. The operation was formally defined as:

(4) $$XOR\;\left\{\vec{B}\right\}=\;\left\{\begin{array}{c} 0\;if\;\sum_{k\;=\;1}^{n}b_{k}\mathrm{is}\;\mathrm{even}\\ 1\;if\;\sum_{k\;=\;1}^{n}b_{k}\mathrm{is}\;\mathrm{odd}. \end{array}\right.$$

The operation returns 0 for an even number of ones in $\vec{B}$, and 1 for an odd number, thus giving information on the parity of a bit sequence of length *n*. For the results shown in Figure 2 of the main text, the n‐bit input (i.e., random binary vector) was multiplied by a maximal phase shift of $\frac{5}{3}\pi$, which was found empirically to yield the lowest overall bit error rate.

*Nonlinear function regression* – The regression of a nonlinear function was seen as the basic functionality of a neuromorphic processor to confirm its ability to adapt to any given function (known as the universal function approximation theorem^[^ ^55^ ^]^). To demonstrate universal function approximation, the normalized $sinc\;\left(x\right)=\frac{sin(x\pi )}{x\pi }\;$ function was used.^[^ ^18^, ^30^ ^]^ For the encoding, a random phase mask (values from 0 to 1) of size 1 × 100, with bins equally distributed in the optical C‐band was implemented. Subsequently, 1000 randomly generated phase values between − π and π were multiplied by the random phase mask and measured the result. Training and testing were performed with equal splitting ratios (i.e., 500 samples each) on 100 spectral bins found via our equal‐distance search algorithm (see Table S1, Supporting Information).

*MNIST* – The dimensionality of the original MNIST data exceeds the available number of inputs for our system. As depicted in Figure S9 (Supporting Information), the original 28 × 28 pixel images were downsampled to 10 × 10 pixels through a bicubic interpolation method. Subsequently, the 2D image data was flattened to generate a one‐dimensional vector of size 1 × 100 and fed the result to the programmable optical filter. The flattening was achieved by a straight‐forward clockwise spiral unwrapping, starting from the image's top‐left corner and ending at the center.

*COVID‐19 audio samples* – The dataset used was available through the INTERSPEECH 2021 ComParE challenge and contains 856 audio samples.^[^ ^44^, ^56^ ^]^

The dataset was initially split into 299 training, 281 verification, and 276 testing data samples. Low‐sampling‐rate recordings were explicitly removed to remove biased data,^[^ ^44^, ^56^ ^]^ which had shown to give significantly improved prediction results, an artifact that had also been confirmed by the challenge holders. To reduce the impact of data imbalance (negative samples ≫ positive samples) and to avoid potential prejudice of the bias‐corrected split, all subsets were first recombined and randomized. It was then split into new training and test sets with a 0.75 training‐to‐testing sample ratio. A custom two‐fold feature extraction method was applied to reduce the input feature size from 6000 samples to only 30 principal frequency components (see Supporting Information, Section 2, paragraph “COVID‐19 data set” and Figure S9, Supporting Information).

To evaluate the performance of an imbalanced data set, such as in the case of our COVID‐19 samples, the default unweighted average recall (UAR) was used as the benchmark value for the INTERSPEECH challenge.^[^ ^44^ ^]^ The UAR was calculated as:

(5) $$UAR\;=\frac{1}{\#\;of\;classes}\;\cdot Sensitivity+\frac{1}{\#\;of\;classes}\cdot Specificity$$

The sensitivity was the fraction of correct (i.e., true) positives divided by the total amount of positive (i.e., true plus false) predictions. The specificity was the fraction of correct (i.e., true) negatives divided by the total number of negative (i.e., true plus false) predictions. UAR values above 0.5 indicate a result better than random guessing. The benchmark value for speech recognition in challenge^[^ ^56^ ^]^ was currently a UAR of 0.709.

### Identification of Artificial Neural Network Primitives

To evaluate the system, shallow, fully connected neural network architecture of minimum complexity, yielding similar performance, were searched for in the n‐bit parity, IRIS, WINE, Abalone, and MNIST tasks. Such network architectures, called primitives, were restricted to a maximum of 3 hidden layers and implemented using the PyTorch library.^[^ ^57^ ^]^ To reduce the solution space, the number of nodes *D*_1_ was iterated in the first layer across an exponentially increasing search grid (i.e., from *D*_1_ = 4, 8, 16, …, to 4096 nodes in the first layer) and followed strict rules in calculating the node number for subsequent layers (i.e., for 2‐layer networks: number of nodes in the 2^nd^ layer *D*_2_ = *D*_1_/8; for 3‐layer networks: *D*_2_ = *D*_1_/4, number of nodes in the 3rd layer *D*_3_ = *D*_2_/2). Finally, the networks with the fewest nodes were chosen and performed similarly to our experimental ELM approach as a task‐specific network primitive. The final configurations and parameters for each network primitive shown in this study are summarized in Table S3 (Supporting Information).

Notably, during the search for the optimal network geometries, the use of the MSE loss metric failed to provide a generalized solution for the Abalone dataset (see Figure S11, Supporting Information). The predicted ages were scattered around the weighted average of 0.3 while still achieving a reasonably low loss value (RMSE = 0.119 for 1024 nodes). To improve the generalization capability, KL‐divergence was implemented as a loss metric (using the PyTorch function *KLDivLoss* ^[^ ^57^ ^]^), which increased the prediction accuracy to a converted loss of RMSE = 0.095 with 128 nodes. It was worth noting that even though an increased performance (and generalization) was achieved with the KL‐divergence metric, the evaluation was more complex and time‐consuming. Furthermore, despite those additional efforts, any primitive architecture that came close to the low error rate of the fiber‐based setup could not be identified. Therefore, the best‐performing neural network was chosen as “primitive” in Figure 3.

For the digital performance comparison of the COVID‐19 data set (Figure S10, Supporting Information), a support vector machine (SVM) model was implemented using the SCIKIT‐LEARN toolkit with the sub‐class LINEARSVC.^[^ ^58^ ^]^ This classifier converged faster and better than any other tested neural network.^[^ ^59^ ^]^ The hyper‐parameter optimization was conducted with a 5‐fold cross‐validation scheme and an *l* ^2^ penalty as the loss function. The optimal penalty parameter C was determined by exhaustively experimenting with values from 10^−5^ to 10, and found 0.05 to be the optimum value.

In all comparisons, the data partitioning (training and test sets) and the pre‐processing of input features were fixed for each data collection set to be the same for both the digital networks and the experiment.

### Definitions on Soliton Fission

Broadband light generation could be achieved by different nonlinear optical effects, such as four‐wave mixing, cross‐ and self‐phase modulation, or soliton fission, depending on the pulse features (power, duration) and waveguide design (dispersion, nonlinearity).^[^ ^26^ ^]^ Soliton fission was the main method for coherent, broadband supercontinuum generation.^[^ ^37^ ^]^ In short, temporal solitons were self‐regulating pulses propagating inside a waveguide due to the interplay of nonlinearity (i.e., Kerr effect) and dispersion. For specific input and waveguide configurations, higher‐order solitons could be generated.^[^ ^26^, ^37^ ^]^ The soliton order *N* could be estimated according to the formula:

(6) $$N^{2}=\frac{T_{0}^{2}\gamma P_{0}}{\left|\beta_{2}\right|}$$

Here, *T_0_*, *P_0_*, *γ*, and *β_2_* denote the 1/e pulse width, the peak power, and the nonlinear and the second‐order dispersion coefficients, respectively. While higher‐order solitons would theoretically maintain their order, perturbations (e.g., third‐order dispersion, Raman) in the experimental realization eventually cause them to break apart into a maximum of *N* fundamental solitons of order *N’  =*  1 during propagation. This splitting, also called soliton fission, was often accompanied by broadband light generation due to the emission of phase‐matched dispersive waves (also known as optical Cherenkov radiation) and various related nonlinear phenomena^[^ ^26^, ^37^ ^]^ This process was coherent, which means reproducible with each optical pulse as long as the order and pulse width of the input soliton remain small (i.e., *N <* 10 and *T_0_ <* 200 fs^[^ ^26^ ^]^).

## Conflict of Interest

The authors declare no conflict of interest.

## Supporting information

Supporting Information

## Data Availability

All data sets used in this work are publicly available from the UCLA machine‐learning repository (IRIS, WINE, Abalone) at https://archive‐beta.ics.uci.edu/. The MNIST dataset was obtained from Kaggle at https://www.kaggle.com/hojjatk/mnist‐dataset. The COVID‐19 speech data set was provided for the INTERSPEECH challenge organized by the University of Cambridge. The inset images of a versicolor orchid, a wine glass, and an Abalone shell in Figure 3 were reused from Wikimedia Commons and fall under the public domain. All measured and simulated data are available in an online repository linked to DOI 10.6084/m9.figshare.21717689. A public version of the analysis codes and network models is available in an online repository linked to this DOI: 10.6084/m9.figshare.21717689.

## References

[1] E. Strubell , A. Ganesh , A. McCallum , in Proceedings of the 57th Annual Meeting of the Association for Computational Linguistics, Stroudsburg, PA, USA, 2019, pp. 3645–3650.

[2] D. V. Christensen , R. Dittmann , B. Linares‐Barranco , A. Sebastian , M. Le Gallo , A. Redaelli , S. Slesazeck , T. Mikolajick , S. Spiga , S. Menzel , I. Valov , G. Milano , C. Ricciardi , S.‐J. Liang , F. Miao , M. Lanza , T. J. Quill , S. T. Keene , A. Salleo , J. Grollier , D. Marković , A. Mizrahi , P. Yao , J. J. Yang , G. Indiveri , J. P. Strachan , S. Datta , E. Vianello , A. Valentian , J. Feldmann , et al., Neuromor. Comput. Engin. 2022, 2, 022501.

[3] G. Wetzstein , A. Ozcan , S. Gigan , S. Fan , D. Englund , M. Soljačić , C. Denz , D. A. B. Miller , D. Psaltis , Nature 2020, 588, 39. 33268862 10.1038/s41586-020-2973-6

[4] B. J. Shastri , A. N. Tait , T. Ferreira de Lima , W. H. P. Pernice , H. Bhaskaran , C. D. Wright , P. R. Prucnal , Nature Photonics 2021, 15, 102.

[5] X. Guo , J. Xiang , Y. Zhang , Y. Su , Adv Photo Res 2021, 2, 2000212.

[6] C. Zuo , Q. Chen , Light Sci Appl 2022, 11, 208. 35794086 10.1038/s41377-022-00903-8 PMC9259600

[7] R. Hamerly , L. Bernstein , A. Sludds , M. Soljačić , D. Englund , Phys. Rev. X 2019, 9, 021032.

[8] X. Xu , M. Tan , B. Corcoran , J. Wu , T. G. Nguyen , A. Boes , S. T. Chu , B. E. Little , R. Morandotti , A. Mitchell , D. G. Hicks , D. J. Moss , Laser Photonics Rev. 2020, 14, 2000070.

[9] J. Feldmann , N. Youngblood , M. Karpov , H. Gehring , X. Li , M. Stappers , M. Le Gallo , X. Fu , A. Lukashchuk , A. S. Raja , J. Liu , C. D. Wright , A. Sebastian , T. J. Kippenberg , W. H. P. Pernice , H. Bhaskaran , Nature 2021, 589, 52. 33408373 10.1038/s41586-020-03070-1

[10] M. Miscuglio , A. Mehrabian , Z. Hu , S. I. Azzam , J. George , A. V. Kildishev , M. Pelton , V. J. Sorger , Opt. Mater. Express 2018, 8, 3851.

[11] W. Wang , S. Gao , Y. Wang , Y. Li , W. Yue , H. Niu , F. Yin , Y. Guo , G. Shen , Adv. Scien. 2022, 9, 2105577. 10.1002/advs.202105577 PMC9534950 35945187

[12] S. Yu , X. Piao , N. Park , Adv. Sci. 2019, 6, 1900771. 10.1002/advs.201900771 PMC6685464 31406676

[13] T. Bu , S. Kumar , H. Zhang , I. Huang , Y.‐P. Huang , Opt. Lett. 2020, 45, 6771. 33325893 10.1364/OL.411564

[14] L. G. Wright , T. Onodera , M. M. Stein , T. Wang , D. T. Schachter , Z. Hu , P. L. McMahon , Nature 2022, 601, 549. 35082422 10.1038/s41586-021-04223-6 PMC8791835

[15] Z. Xu , B. Tang , X. Zhang , J. F. Leong , J. Pan , S. Hooda , E. Zamburg , A. V. Thean , Light Sci Appl 2022, 11, 288. 36202804 10.1038/s41377-022-00976-5 PMC9537414

[16] M. A. Al‐Qadasi , L. Chrostowski , B. J. Shastri , S. Shekhar , APL Photonics 2022, 7, 020902.

[17] T. W. Hughes , I. A. D. Williamson , M. Minkov , S. Fan , Sci. Adv. 2019, 5, eaay6946. 31903420 10.1126/sciadv.aay6946 PMC6924985

[18] G. Marcucci , D. Pierangeli , C. Conti , Phys. Rev. Lett. 2020, 125, 093901. 32915624 10.1103/PhysRevLett.125.093901

[19] M. Nakajima , K. Tanaka , T. Hashimoto , IEEE Transactions on Neural Networks and Learning Systems 2021, 2686. 10.1109/TNNLS.2021.3120472 34731081

[20] W. Maass , T. Natschläger , H. Markram , Neural Computat. 2002, 14, 2531. 10.1162/089976602760407955 12433288

[21] M. Rabinovich , R. Huerta , G. Laurent , Science 2008, 321, 48. 18599763 10.1126/science.1155564

[22] C. Fernando , S. Sojakka , in Advances in Artificial Life (Eds: W. Banzhaf , J. Ziegler , T. Christaller , P. Dittrich , J.T. Kim ), Springer, Berlin Heidelberg 2003, 2801, pp. 588–597.

[23] I. S. Maksymov , A. Pototsky , S. A. Suslov , Phys. Rev. E 2022, 105, 044206. 35590644 10.1103/PhysRevE.105.044206

[24] U. Tegin , M. Yildirim , I. Oguz , C. Moser , D. Psaltis , Nat. Computat. Scien. 2021, 1, 542. 10.1038/s43588-021-00112-0 38217249

[25] C. Hager , H. D. Pfister , IEEE J. Select. Areas Commun. 2021, 39, 280.

[26] J. M. Dudley , G. Genty , S. Coen , Rev. Mod. Phys. 2006, 78, 1135.

[27] B. Wetzel , M. Kues , P. Roztocki , C. Reimer , P. L. Godin , M. Rowley , B. E. Little , S. T. Chu , E. A. Viktorov , D. J. Moss , A. Pasquazi , M. Peccianti , R. Morandotti , Nature Communications 2018, 9, 4884. 10.1038/s41467-018-07141-w PMC6244003 30459363

[28] T. Zhou , F. Scalzo , B. Jalali , J. Lightwave Technol. 2022, 40, 1308.

[29] C. M. Bishop , Machine Learning and Pattern Recognition, Springer Science and Business Media LLC, New York 2007.

[30] G. B. Huang , Q. Y. Zhu , C. K. Siew , in IEEE International Conference on Neural Networks, 2004, 2, pp. 985–990.

[31] A. Bendahmane , F. Braud , M. Conforti , B. Barviau , A. Mussot , A. Kudlinski , Optica 2014, 1, 243. 10.1364/OE.22.025673 25401600

[32] X. Qi , R. Scheibinger , J. Nold , S. Junaid , M. Chemnitz , M. A. Schmidt , APL Photonics 2022, 7, 116106.

[33] T. A. K. Lühder , M. Chemnitz , H. Schneidewind , E. P. Schartner , H. Ebendorff‐Heidepriem , M. A. Schmidt , Advanced Science 2022, 9, 2103864. 35038237 10.1002/advs.202103864 PMC8922130

[34] I. Oguz , J.‐L. Hsieh , N. U. Dinc , U. Teğin , M. Yildirim , C. Gigli , C. Moser , D. Psaltis , Programming Nonlinear Propagation for Efficient Optical Learning Machines , 2022, arXiv 2208.04951.

[35] D. Brunner , M. C. Soriano , C. R. Mirasso , I. Fischer , Nat. Commun. 2013, 4, 1364. 23322052 10.1038/ncomms2368 PMC3562454

[36] M. Lukoševičius , H. Jaeger , B. Schrauwen , Künstl Intell 2012, 26, 365.

[37] A. V. Husakou , J. Herrmann , Phys. Rev. Lett. 2001, 87, 203901. 11690475 10.1103/PhysRevLett.87.203901

[38] L. Salmela , M. Hary , M. Mabed , A. Foi , J. M. Dudley , G. Genty , Opt. Lett. 2022, 47, 802. 35167529 10.1364/OL.448571

[39] M. Minsky , S. A. Papert , Perceptrons: An Introduction to Computational Geometry, The MIT Press, Cambridge 1969.

[40] M. S. Advani , A. M. Saxe , H. Sompolinsky , Neural Netw. 2020, 132, 428. 33022471 10.1016/j.neunet.2020.08.022 PMC7685244

[41] Y. LeCun , L. Bottou , Y. Bengio , P. Haffner , Proceedings of the IEEE 1998, 86, 2278.

[42] D. Pierangeli , G. Marcucci , C. Conti , Photon. Res. 2021, 9, 1446.

[43] P. Antonik , N. Marsal , D. Rontani , IEEE J. Sel. Top. Quantum Electron. 2020, 26, 1. 10.1109/JSTQE.2019.2921376 PMC7677864 33223801

[44] B. W. Schuller , A. Batliner , C. Bergler , C. Mascolo , J. Han , I. Lefter , H. Kaya , S. Amiriparian , A. Baird , L. Stappen , S. Ottl , M. Gerczuk , P. Tzirakis , C. Brown , J. Chauhan , A. Grammenos , A. Hasthanasombat , D. Spathis , T. Xia , P. Cicuta , L. J. M. Rothkrantz , J. Zwerts , J. Treep , C. Kaandorp , INTERSPEECH 2021 Comput. Paralingu. Challenge COVID‐19 2021, arXiv 2102.13468.

[45] Y. Fang , C. Bao , S. Li , Z. Wang , W. Geng , Y. Wang , X. Han , J. Jiang , W. Zhang , Z. Pan , Z. Li , Y. Yue , Laser Photonics Rev. 2023, 17, 2200205.

[46] H. Zia , K. Ye , Y. Klaver , D. Marpaung , K.‐J. Boller , Adv. Photon. Res. 2023, 4, 2200296.

[47] T. Sylvestre , E. Genier , E. Genier , A. N. Ghosh , A. N. Ghosh , P. Bowen , G. Genty , J. Troles , A. Mussot , A. C. Peacock , M. Klimczak , A. M. Heidt , J. C. Travers , O. Bang , O. , J. M. Dudley , J. Opt. Soc. Am. B 2021, 38, F90.

[48] M. Chemnitz , S. Junaid , M. A. Schmidt , Laser Photonics Rev. 2023, 17, 2300126.

[49] C. Markos , J. C. Travers , A. Abdolvand , B. J. Eggleton , O. Bang , Rev. Mod. Phys. 2017, 89, 045003.

[50] M. Yildirim , I. Oguz , F. Kaufmann , M. R. Escale , R. Grange , D. Psaltis , C. Moser , Nonlin. Optical Data Transf. Mach. Learn 2022, arXiv 2208.09398.

[51] D. Markovic , A. Mizrahi , D. Querlioz , J. Grollier , Nature Reviews Physics 2020, 2, 499.

[52] A. Mahjoubfar , D. V. Churkin , S. Barland , N. Broderick , S. K. Turitsyn , B. Jalali , Nat. Photonics 2017, 11, 341.

[53] F. G. Omenetto , A. J. Taylor , M. D. Moores , D. H. Reitze , Opt. Lett. 2001, 26, 938. 18040498 10.1364/ol.26.000938

[54] M. Stone , J. R. Stat. Soc., B: Stat. Methodol. 1974, 36, 111.

[55] K. Hornik , M. Stinchcombe , H. White , Neural Networks 1989, 2, 359.

[56] H. Coppock , A. Akman , C. Bergler , M. Gerczuk , C. Brown , J. Chauhan , A. Grammenos , A. Hasthanasombat , D. Spathis , T. Xia , P. Cicuta , J. Han , S. Amiriparian , A. Baird , L. Stappen , S. Ottl , P. Tzirakis , A. Batliner , C. Mascolo , B. W. Schuller , A Summ. ComParE COVID‐19 Challeng 2022, arXiv 2202.08981.

[57] A. Paszke , S. Gross , F. Massa , A. Lerer , J. Bradbury , G. Chanan , T. Killeen , Z. Lin , N. Gimelshein , L. Antiga , A. Desmaison , A. Köpf , E. Yang , Z. DeVito , M. Raison , A. Tejani , S. Chilamkurthy , B. Steiner , L. Fang , J. Bai , S. P. y. T. Chintala , An Imperative Style, High‐Performance Deep Learning Library . 2019, arXiv 2019, 1912.01703.

[58] F. Pedregosa , G. Varoquaux , A. Gramfort , V. Michel , B. Thirion , O. Grisel , M. Blondel , A. Müller , J. Nothman , G. Louppe , P. Prettenhofer , R. Weiss , V. Dubourg , J. Vanderplas , A. Passos , D. Cournapeau , M. Brucher , M. Perrot , É. S.‐L. Duchesnay , Mach. Learn. Python 2012, arXiv 1201.0490.

[59] Y. Zhu , T. H. Falk , in ICASSP 2022 –2022 IEEE International Conference on Acoustics, Speech and Signal Processing (ICASSP), IEEE, Singapore 2022, pp. 8997–9001, 10.1109/ICASSP43922.2022.9746471.

