# Supplementary material for: Neuromorphic Computing via Fission‐based Broadband Frequency Generation
Source: Adv Sci (Weinh). 2023 Oct 2;10(35):2303835. doi: 10.1002/advs.202303835 (PMC10724387; doi:10.1002/advs.202303835)
Supplement: Supplementary file 1 — Supporting Information [file ADVS-10-2303835-s001.pdf]

ADVANCED SCIENCE
Open Access

Supporting Information

for *Adv. Sci.*, DOI 10.1002/advs.202303835

Neuromorphic Computing via Fission-based Broadband Frequency Generation

Bennet Fischer, Mario Chemnitz, Yi Zhu, Nicolas Perron, Piotr Roztocki, Benjamin MacLellan, Luigi Di Lauro, A. Aadhi, Cristina Rimoldi, Tiago H. Falk and Roberto Morandotti**

Supplementary Materials for

Neuromorphic Computing via Fission-based Broadband Frequency Generation

Bennet Fischer[1,2,†], Mario Chemnitz[1,2,†,*], Yi Zhu[1], Nicolas Perron[1], Piotr Roztocki[1,3], Benjamin MacLellan[1], Luigi Di Lauro[1], A. Aadhi[1], Cristina Rimoldi[1,4], Tiago H. Falk[1], Roberto Morandotti[1,*]

[1]Institut National de la Recherche Scientifique - Énergie, Matériaux et Télécommunications; 1650 Blvd. Lionel-Boulet, J3X 1S2 Varennes, Quebec, Canada.

[2]Leibniz Institute of Photonic Technology; Albert-Einstein Str. 9, 07745 Jena, Germany.

[3]Ki3 Photonics Technologies, 2547 Rue Sicard, H1V 2Y8 Montreal, Quebec, Canada.

[4]Dipartimento di Elettronica e Telecomunicazioni, Politecnico di Torino, Corso Duca degli Abruzzi 24, 10129 Torino, Italy.

*Corresponding authors. Email: mario.chemnitz@leibniz-ipht.de, roberto.morandotti@inrs.ca

[†] These authors contributed equally to this work.

Table of Contents

Supplementary Text
- A. Mathematical analogy between neural networks and nonlinear pulse propagation 2
- B. Phase sensitivity of soliton-fission-based neural emulation 3
- C. Training results 4
- D. Optical neural network performance comparison 6
- E. Parameters of identified neural network primitives 6
- F. Artificial neural network performance on the *n*-bit parity operation 6
- G. Performance and energy considerations 7

References 8

Figures 10

Tables 21

Supplementary Text

A. Mathematical analogy between neural networks and nonlinear pulse propagation

In general, nonlinear pulse propagation in optical waveguides, including soliton fission, can be modeled by means of the generalized nonlinear Schrödinger equation (GNLSE) in the time domain[1]:

$$\frac{\partial}{\partial z}A - \sum_{k\geq 2}\frac{i^{k+1}}{k!}\beta_k\frac{\partial^k}{\partial T^k}A = i\gamma\left(1 + i\tau_{shock}\frac{\partial}{\partial T}\right)(R * |A|^2)A \quad \text{(S1)}$$

Here, $A(z, T)$ is the pulse envelope in a comoving frame $T = t - \beta_1 z$ at the group velocity $1/\beta_1$. The parameters β_k and γ denote the higher-order dispersion terms and the nonlinear parameter of the waveguide, respectively. τ_{shock} is the self-steepening parameter and $R(T)$ accounts for the nonlinear response of the material (including instantaneous electronic and non-instantaneous Raman contribution)[2]. The * operator substitutes a convolution operation of the form $R * |A|^2 = \int_{-\infty}^{\infty} R(T')|A(z, T - T')|^2\, dT'$.

The GNLSE is a partial differential equation without analytical solutions and requires numerical approximation methods to be solved, such as the split-step Fourier algorithm. The iterative split-step Fourier method can be applied to differential equations of the form:

$$\frac{\partial}{\partial z}A(z,T) = \hat{\mathcal{D}}A + \hat{\mathcal{N}}A, \quad \text{(S2)}$$

with the propagation coordinate z, the optical field envelope A, the linear dispersion operator $\hat{\mathcal{D}}$, and the nonlinear operator $\hat{\mathcal{N}}$. In the numeric split-step approach[2], the linear and nonlinear terms of the equation are commonly solved in the frequency and time domains, respectively, in an alternate sequence mediated by the Fourier transform (FT). Simulations in this work have been performed by solving Eq. (S1) numerically in this way, where the nonlinear step has been performed using a 4th-order Runge-Kutta integrator to resolve the z derivation.

Under certain assumptions, it can be shown that a discretized nonlinear Schrödinger equation displays the same mathematical form of a feed-forward artificial neural network. Without loss of generality, we may neglect the weak molecular Raman response of the waveguide (i.e., $R(T) \approx \delta(T) \Longrightarrow R * |A|^2 \approx |A|^2$, where $\delta(x)$ is the Kronecker delta function) and assume a non-dispersive nonlinear material response (i.e., $\tau_{shock} = 0$) in first approximation. Those assumptions reduce Eq. (S1) to the specialized nonlinear Schrödinger equation of the form

$$\frac{\partial}{\partial z}A = \sum_{k\geq 2}\frac{i^{k+1}}{k!}\beta_k\frac{\partial^k}{\partial T^k}A + i\gamma|A|^2A \quad \text{(S3)}$$

In the framework of the split-step Fourier method, we solve the linear part of Eq. (S3) separately from the nonlinear part. The linear equation $\partial_z A(T; z) = \hat{\mathcal{D}}A$ has an analytic solution in the Fourier space, which can be expressed as:

$$A(T; \Delta z) = FT^{-1}\{\tilde{A}(\Omega; \mathrm{z} = 0)e^{i\beta(\Omega)\Delta z}\}, \quad \text{(S4)}$$

where $\tilde{A} = FT\{A\}$ is the optical field amplitudes in the frequency domain, $\beta = \sum_{k\geq 2}\frac{1}{k!}\beta_k\Omega^k$ is the propagation constant, Ω is the angular frequency difference with respect to the central frequency,

and Δz is the step-size. We find that the solution in Eq. (S4) is a multiplication in the frequency domain, which can be formally written as a convolution in the time domain

$$A(T;\Delta z) = \int A(T';0)\mathcal{D}(T-T')\,dT' \,, \quad \text{(S5)}$$

where $\mathcal{D}(T) = FT^{-1}\{e^{\frac{i\beta(\Omega)\Delta z}{2}}\}(T)$ is the temporal impulse response of the waveguide over half a propagation step $\Delta z/2$. Half steps are the convention used in the split-step algorithm (see Eq. (S7) below). Discretizing the time to a grid with steps $p\Delta T = T_p$ and the propagation axis to a grid with steps $k\Delta z = z_k$, allows us to approximate Eq. (S5) per half propagation step with

$$A_p^{k+1/2} = \sum_q \mathcal{D}_{pq} A_q^k \,, \quad \text{(S6)}$$

where $A_p^k = A(p\Delta T; k\Delta z)$ and $\mathcal{D}_{pq} = \mathcal{D}((p-q)\Delta T)\Delta T$.

We note that Eq. (S6) has a formal resemblance to a sum & weight operation, i.e., the linking function of a neural network layer. Here, the dispersive coefficients $\mathcal{D}_{pq}$ correspond to the temporal impulse response of the waveguide shifted in time relative to the temporal field components A_q^k. The coefficients act as static, waveguide-specific weights on the complex-valued field amplitudes[3].

On the other hand, the nonlinear term of Eq. (S3), i.e., $\partial_z A(z,T) = i\gamma|A|^2 A = \widehat{N}(A)$, can be solved straightforwardly by discretizing the derivation in z. For simplicity, we demonstrate this using the Euler method $\partial_z A \approx \frac{1}{\Delta z}(A_p^{k+1} - A_p^k)$. Hence, the kth nonlinear step along Δz can be approximated in the time domain by means of a simplified form, such as:

$$A_p^k = i\gamma\Delta z\left|A_p^{k-1/2}\right|^2 A_p^{k-1/2} + A_p^{k-1} = \mathcal{N}(A_p^{k-1/2}) \,, \quad \text{(S7)}$$

Eq. (S7) resembles a cubic activation function of the temporal field amplitudes A_p^k, mediated by a nonlinear gain parameter γ. The activation potential $\left|A_p^k\right|^2$ may be the pump field itself (i.e., self-activation) or other generated fields that temporally overlap with the main field (i.e., cross-activation). Inserting Eq. (S6) into Eq. (S7) yields an equation that formally matches the mathematical model of artificial neural nodes in a network (cf. Eq. (2) in the main text).

We stress that, beyond a common stability criterion, step size Δz can be chosen to be arbitrarily small. Thus, it has no practical meaning to estimate the computation advantage of neuromorphic wave computing by counting the mathematical operations needed to obtain a stable solution of Eq. (S7) over the entire fiber length.

B. Phase sensitivity of soliton-fission-based neural emulation

To investigate the phase dependency and sensitivity of the system, we performed a straightforward single-bit-shift experiment. We scanned a single encoding bit (width = 1 nm) through the available bandwidth of the programmable filter (1528 nm to 1600 nm), with a step size of 2.0 nm. For each bit position, we collected the output spectrum of the HNLF for four different phase factors. This simple yet informative experiment reveals the high phase sensitivity of the soliton fission dynamics to phase information at the input. Fig. S3 shows the upper and lower intensity bounds across the readout bandwidth, which can be understood as a measure of the system's maximum readout dynamics for a chosen phase factor.

Even for small phase values (0.1π), intensity variations are visible in the output spectra (up to 5 dB). With increasing phase factors (0.4π, 0.7π, 1.0π), variations increase, thus indicating a higher susceptibility to changes in the bit position. While one would usually assume better system performance for larger output variations, in our experiments we found that phase factors of around $\frac{\pi}{8}$ performed best in all tested benchmarks, independently of the input feature size (i.e., the phase information per encoding wavelength).

C. Training results

Sinc regression – In our implementation, we repeatably measured a random bit string with 1000 random phase values. Notably, we avoided regularization (i.e., ridge regression) in the learning process, which appears to be a common, yet more complex approach to achieve higher fitting accuracies. Fig. S4 shows the results for the Sinc regression. We obtained a test (training) RMSE of 0.0175 (3.05×10^{-4}). A similar approach based on spatial modes[4] requires two orders of magnitude higher pulse energy (320 nJ) than our frequency-based approach (89 pJ). Yet, it exhibits a higher error rate (RMSE of 0.067) for this task. The spatial mode implementation also reported performance limitations due to modal self-cleaning effects with higher powers. In comparison, we have not observed such power restrictions in our experiments, and we do not expect such limiting mechanisms in soliton fission-driven nonlinear dynamics.

n-bit parity – Fig. S5 depicts the training results from Fig. 2(G) in the main text. We define the system's operation fidelity as the accuracy of a class prediction problem in machine learning. It is a measure of how well a model correctly identifies or excludes a condition. In this context, accuracy is defined as the proportion of true results (both true positives (TP) and true negatives (TN)) in the total number of samples examined:

$$Accuracy = \frac{\#\ of\ TP + \#\ of\ TN}{\#\ of\ samples}. \qquad (S8)$$

Like the test results, the training results improve operation fidelity with increasing system nonlinearity (soliton number). We further find that the operation performance can be scaled with the amount of chosen readout bins, offering an additional control parameter to our approach. For bit lengths from $n = 4$ to $n = 7$ bits, we studied the operation fidelity for increasing readout bins from 4 and 7 to 125 bins. Notably, the observed improvement in performance with increasing system nonlinearity is independent of the chosen number of read-outs observed for a fixed number of read-out bins (e.g., 7 read-outs for bit length n = 4 to 7). The remaining training parameters are specified in Table S1. and Fig. S6, which present the results for two different input power configurations (i.e., soliton numbers $N = 2$ and $N = 5$). The results show that increased readouts beyond the input feature size (i.e., bit length n) can drastically increase system performance, essentially serving as a free system hyperparameter.

On the other hand, with a higher number of spectral bins, we observed effects of overfitting (i.e., a decrease or oscillation of test fidelities when we reach a training accuracy close to 100%), imposing a practical limitation to the system confidence bounds. Notably, the bin sweep confirms the overall trend of the results shown in Fig. 2. In particular, with a higher soliton number, operation fidelity can be improved for a constant number of readout bins.

Task-dependent benchmarks - The confusion matrices of the training set for all task-dependent benchmarks are shown in Fig. S7 and complement the test results in Fig. 3.

MNIST data set – The recognition of handwritten digits was the most challenging task for our implementation. We used the MNIST set to study the dependence of the system performance on (i) the maximum phase factor of the encoding, (ii) the spectral resolution of the read-out, and (iii) the number of readout bins. The results in Fig. S8(A) show a slight decrease in prediction accuracy for phase factors, increasing from $\frac{\pi}{128}$ to $\frac{\pi}{8}$, independently of the chosen read-out bin number.

Subsequently, we investigated the performance dependence on the number of readout bins similarly to the n-bit parity study (Fig. S8(C), for a phase factor of $\frac{\pi}{8}$ and 0.5 nm resolution). In agreement with previous findings, we observed that increasing the number of readout bins improves system performance up to a certain limit. Beyond ~100 readout bins, the consequences of overfitting became apparent (i.e., further decrease in the training error but stagnation in the test error).

Finally, we observed that system performance also dropped with decreasing spectral resolution (Fig. S8(B)), and the best results were achieved with the highest resolution available (0.125 nm).

It is important to note that while an increase in resolution improves system performance (more available spectral combinations), it also drastically impacts the measurement time per inference in the current platform implementation due to characteristics of the used spectrometer. The measurement time per scan increases from seconds (in the case of 2 nm resolution) to minutes (in the case of <0.5 nm resolution). At the highest resolution (0.125nm), we achieved the best system performance, however, this setting led to impractical measurement times per sample (~2mins). Thus, we settled for a compromise between system performance and total acquisition time by choosing a 0.5 nm spectral resolution for the final acquisition of the MNIST data set (approx. 1 min. acquisition time per sample).

COVID-19 data set – To ensure a fair comparison between our implementation and the digital support vector machine (SVM) approach, the same training and test sets were used for both. We used a 5-fold cross-validation during training for the software SVM implementation to determine the correct hyperparameters. Specifically, for the SVM we used a penalty parameter of 0.05, and an l^2 penalty as the loss function[5].

The benchmark feature set used in the challenge contains over 6000 features extracted from the widely used openSMILE toolbox . To provide helpful input information to our system (i.e., a feature size $\ll$400), we performed a two-step pre-processing on the raw audio data to obtain 30 principal features, which were eventually given as inputs to both the SVM software classifier and our experimental system. The method for feature reduction is illustrated in Fig. S9.

Our technique builds on creating a modulation spectrogram as the basis for a two-fold feature extraction scheme. Such an approach is quite new and has outperformed conventional spectrogram-based features for COVID-19 detection using SVMs[7]. A modulation spectrogram describes the rate of change for the individual kilohertz-rate frequency components and is particularly useful for analyzing highly dynamic, time-variant data[8]. We first transformed the

initial audio signal $x(t)$ into a 2-dimensional time-frequency space ($X(T,f)$) using a short-time Fourier transform (STFT, 256-point Fast-Fourier transform, FFT). Then, we applied a second FFT on the data per frequency channel along the time-axis[8], resulting in the modulation spectrogram $\chi(f_{mod}, f)$. In our process, we divided the resulting spectrogram into twenty 1 Hz bins on the modulation axis, f_{mod}, and twenty 400 Hz bins on the conventional frequency axis, f, resulting in a 20×20 frequency-frequency map ($\chi_{bin}(f_{mod}, f)$). Next, we performed an initial feature extraction across each dimension (f and f_{mod}), where eight spectral descriptors, such as entropy, centroid, and kurtosis[7] were computed, resulting in an additional 320 low-level descriptors (LLDs, i.e., 2 parameters × 20 bins × 8 descriptors). This first feature extraction step leads to a total of 720 features (400 energies from the binned modulation spectrogram plus the 320 LLDs) that have been successfully applied to COVID-19 detection in recent demonstrations[7]. To further decrease the dimensionality of the input data, we used a second feature extraction in the form of a principal component analysis (PCA) to reduce the feature size to 30.

The achieved results for the COVID-19 classification task from the main text are shown in Fig. S10.

D. Optical Neural Network Performance Comparison

To contextualize our system performance data, we compared different optical neuromorphic processors for the same tasks as shown in Table S2.

E. Parameters of Identified Artificial Neural Network Primitives

Table S3 summarizes the hyper-parameters and achieved performance of the neural network primitives used for the comparison in Fig. 3. We trained each model by means of the stochastic gradient descent (SGD) optimizer with momentum 0.9. We employed a training batch size of 16, which was consistent for all tasks.

F. Artificial Neural Network Performance on the *n*-bit Parity Operation

Numerous studies in computer science demonstrated the dependence of bit length *n* on the network complexity (i.e., more nodes or layers) required to perform the *n*-bit parity operation. Since those models are often based on numerous assumptions about network structure (e.g., direct input-output connections), we aimed to support the claim about increasing network complexity vs. bit length with our own approach, to reveal network primitives. We have studied the operation fidelity for the same bit lengths used in the experiment (i.e., $n = 2$ to $n = 7$) for an increasing number of nodes (2 to 128) in a single-hidden-layer network. Our results in Fig. S11 show that operation fidelity improves significantly with larger networks for a given bit length. This observation holds true for all tested bit lengths. In addition, starting from 5-bit and higher, we observed overfitting effects for a larger number of hidden-layer nodes, ultimately requiring more complex and optimized neural network topologies for robust operation as the bit length increases.

G. Performance and Energy Considerations

Different performance metrics are commonly used to assess the computational performance of hardware. Among the most popular for neural network applications are multiply-and-accumulate (MAC) or floating-point (FLOP) operations, which directly refer to the vector matrix multiplications in neural networks. However, a direct and reliable calculation of such metrics is often not feasible for analog and neuromorphic hardware implementations. A recent suggestion to estimate the computational performance of analog systems builds on the sum of mathematical operations required to simulate the system[9]. The proposed method yields almost arbitrary performance for wave-based computing systems depending on the chosen simulation grid size, which can be adapted and does not relate to the actual neuromorphic task the system has to solve.

Therefore, to compare the computational cost of our approach per given task, we introduce the use of neural network primitives with equivalent task performances as depicted in Fig. 3. Then, to compare the system responses, we calculated the FLOPs carried out by the network primitives using the following method. For a single inference step, the input matrix is of size $[1, D_{in}]$. Here, D_{in} is the dimension of the input vector multiplied for a hidden layer matrix of size $[D_{in}, D_h]$, where D_h is the number of nodes in the hidden layer. The output is then multiplied with another matrix $[D_h, D_{out}]$ to get the final output. Here, D_{out} represents the dimension of the final prediction.

For the first matrix operation, the required FLOPs can be calculated as: $1 \times D_{in} \times D_h + D_h$. For the second matrix operation, the required FLOPs can be calculated as: $1 \times D_h \times D_{out} + D_{out}$. The results for all tasks are summarized in Table S4. E.g., for the IRIS task (see Table S4), the required FLOPs are determined as:

$$FLOP\ (input \rightarrow hidden) = 1 \times 4 \times 4 + 4 = 20$$
$$FLOP\ (hidden \rightarrow output) = 1 \times 4 \times 3 + 3 = 15$$
$$FLOP\ (all) = 35$$

For the SVM classifier we used in the COVID-19 task, the FLOPs of a single inference step can be easily calculated as $2 \times Feature\ Size$ (i.e., 30 in the present study). Therefore, our SVM requires 60 FLOPs to perform the COVID-19 classification. This simplicity is because a linear SVM has a prediction complexity of $O(d)$, where d is the input dimension size, given that there is only a single inner product[5].

To allow for a fair comparison of our experiments with all software classifiers, we exclusively consider the required FLOPs from the input to the hidden layers (i.e., not to the output layer). This is because the readout of our current fiber-based implementation still requires measuring the average value over thousands of optical pulses. In a future system, however, an individual inference could be measured on a single-shot basis (i.e., at 200 MHz rate), e.g., using a recently reported high-speed Waveshaper alternative based on acousto-optic modulators[10] for encoding combined with dispersive Fourier transform and ultrafast detection[11]. Yet, such single-shot implementation would be highly dependent on the chosen components. Hence, in any case, we

cannot provide a trustworthy estimate for the energy consumption of the read-out layer at the current stage.

Table S4 shows the energy comparison between our experimental wave-based approach (neglecting encoding & decoding electronics) and its digital counterparts for one inference step. For comparison, we assume an NVIDIA Kepler K20m GPU with ~50 pJ/FLOP[12].

Interestingly, let's consider highly efficient computing architecture (1 pJ/FLOP[13]). The implemented digital approaches are more energy efficient for inference than the fiber-based system, but only for certain tasks, e.g., IRIS or COVID-19. Notably, this optimized architecture usually operates at reduced bit depths (among other simplifications), which may lead to overall reduced accuracy for universal task applications.

Finally, to better understand our implemented system, we compare the inference energy requirements with similar wave-based processors in Table S5.

References

(1) Agrawal, G. P. Nonlinear Pulse Propagation. In *Nonlinear Fiber Optics*; Academic Press: Boston, 2013; pp 27–56.

(2) Dudley, J. M.; Genty, G.; Coen, S. Supercontinuum Generation in Photonic Crystal Fiber. *Reviews of Modern Physics* **2006**, *78* (4), 1135–1184. https://doi.org/10.1103/RevModPhys.78.1135.

(3) Nakajima, M.; Tanaka, K.; Hashimoto, T. Neural Schrödinger Equation: Physical Law as Deep Neural Network. *IEEE Transactions on Neural Networks and Learning Systems* **2021**, 1–15. https://doi.org/10.1109/TNNLS.2021.3120472.

(4) Tegin, U.; Yildirim, M.; Oguz, I.; Moser, C.; Psaltis, D. Scalable Optical Learning Operator. *Nature Computational Science* **2021**, *1* (8), 542–549. https://doi.org/10.1038/s43588-021-00112-0.

(5) Pedregosa, F.; Varoquaux, G.; Gramfort, A.; Michel, V.; Thirion, B.; Grisel, O.; Blondel, M.; Müller, A.; Nothman, J.; Louppe, G.; Prettenhofer, P.; Weiss, R.; Dubourg, V.; Vanderplas, J.; Passos, A.; Cournapeau, D.; Brucher, M.; Perrot, M.; Duchesnay, É. Scikit-Learn: Machine Learning in Python. *arXiv* **2012**, 1201.0490.

(6) Eyben, F.; Wöllmer, M.; Schuller, B. Opensmile. In *Proceedings of the international conference on Multimedia - MM '10*; ACM Press: New York, New York, USA, 2010; p 1459. https://doi.org/10.1145/1873951.1874246.

(7) Zhu, Y.; Falk, T. H. Fusion of Modulation Spectral and Spectral Features with Symptom Metadata for Improved Speech-Based Covid-19 Detection. In *ICASSP 2022 - 2022 IEEE International Conference on Acoustics, Speech and Signal Processing (ICASSP)*; IEEE, 2022; pp 8997–9001. https://doi.org/10.1109/ICASSP43922.2022.9746471.

(8) Falk, T. H.; Chan, W. Y.; Sejdic, E.; Chau, T. Spectro-Temporal Analysis of Auscultatory Sounds. In *New Development in Biomedical Engineering*; Campolo, D., Ed.; IntechOpen, 2010; pp 93–104.

(9) Wright, L. G.; Onodera, T.; Stein, M. M.; Wang, T.; Schachter, D. T.; Hu, Z.; McMahon, P. L. Deep Physical Neural Networks Trained with Backpropagation. *Nature* **2022**, *601* (7894), 549–555. https://doi.org/10.1038/s41586-021-04223-6.

(10) Liu, X.; Braverman, B.; Boyd, R. W. Using an Acousto-Optic Modulator as a Fast Spatial Light Modulator. *Opt. Express* **2023**, *31* (2), 1501. https://doi.org/10.1364/OE.471910.

(11) Godin, T.; Sader, L.; Khodadad Kashi, A.; Hanzard, P.-H.; Hideur, A.; Moss, D. J.; Morandotti, R.; Genty, G.; Dudley, J. M.; Pasquazi, A.; Kues, M.; Wetzel, B. Recent Advances on Time-Stretch Dispersive Fourier Transform and Its Applications. *Advances in Physics: X* **2022**, *7* (1). https://doi.org/10.1080/23746149.2022.2067487.

(12) Kurowski, K.; Ciznicki, M.; Weglarz, J. Energy Efficiency and Performance Modeling of Stencil Applications on Manycore and GPU Computing Resources. In *2020 20th IEEE/ACM International Symposium on Cluster, Cloud and Internet Computing (CCGRID)*; IEEE, 2020; pp 232–241. https://doi.org/10.1109/CCGrid49817.2020.00-70.

(13) Horowitz, M. Computing's Energy Problem (and What We Can Do about It). In *2014 IEEE international solid-state circuits conference digest of technical papers (ISSCC)*; IEEE, 2014; pp 10–14. https://doi.org/10.1109/ISSCC.2014.6757323.
(14) Zhang, H.; Gu, M.; Jiang, X. D.; Thompson, J.; Cai, H.; Paesani, S.; Santagati, R.; Laing, A.; Zhang, Y.; Yung, M. H.; Shi, Y. Z.; Muhammad, F. K.; Lo, G. Q.; Luo, X. S.; Dong, B.; Kwong, D. L.; Kwek, L. C.; Liu, A. Q. An Optical Neural Chip for Implementing Complex-Valued Neural Network. *Nature Communications* **2021**, *12* (1), 457. https://doi.org/10.1038/s41467-020-20719-7.
(15) Wang, R.; Wang, P.; Luo, G.; Yu, H.; Zhou, X.; Zhang, Y.; Pan, J. Silicon-Based Optical Neural Network Chip Based on Coherent Detection. In *Asia Communications and Photonics Conference/International Conference on Information Photonics and Optical Communications 2020 (ACP/IPOC)*; OSA: Washington, D.C., 2020; p T2D.4. https://doi.org/10.1364/ACPC.2020.T2D.4.
(16) Lupo, A.; Butschek, L.; Massar, S. Photonic Extreme Learning Machine Based on Frequency Multiplexing. *Optics Express* **2021**, *29* (18), 28257. https://doi.org/10.1364/OE.433535.
(17) Lupo, A.; Massar, S. Parallel Extreme Learning Machines Based on Frequency Multiplexing. *Applied Sciences* **2021**, *12* (1), 214. https://doi.org/10.3390/app12010214.
(18) Pierangeli, D.; Marcucci, G.; Conti, C. Photonic Extreme Learning Machine by Free-Space Optical Propagation. *Photonics Research* **2021**, *9* (8), 1446. https://doi.org/10.1364/PRJ.423531.
(19) Tegin, U.; Yildirim, M.; Oguz, I.; Moser, C.; Psaltis, D. Scalable Optical Learning Operator. *Nature Computational Science* **2021**, *1* (8), 542–549. https://doi.org/10.1038/s43588-021-00112-0.
(20) Yildirim, M.; Oguz, I.; Kaufmann, F.; Escale, M. R.; Grange, R.; Psaltis, D.; Moser, C. Nonlinear Optical Data Transformer for Machine Learning. *arXiv* **2022**, 2208.09398.

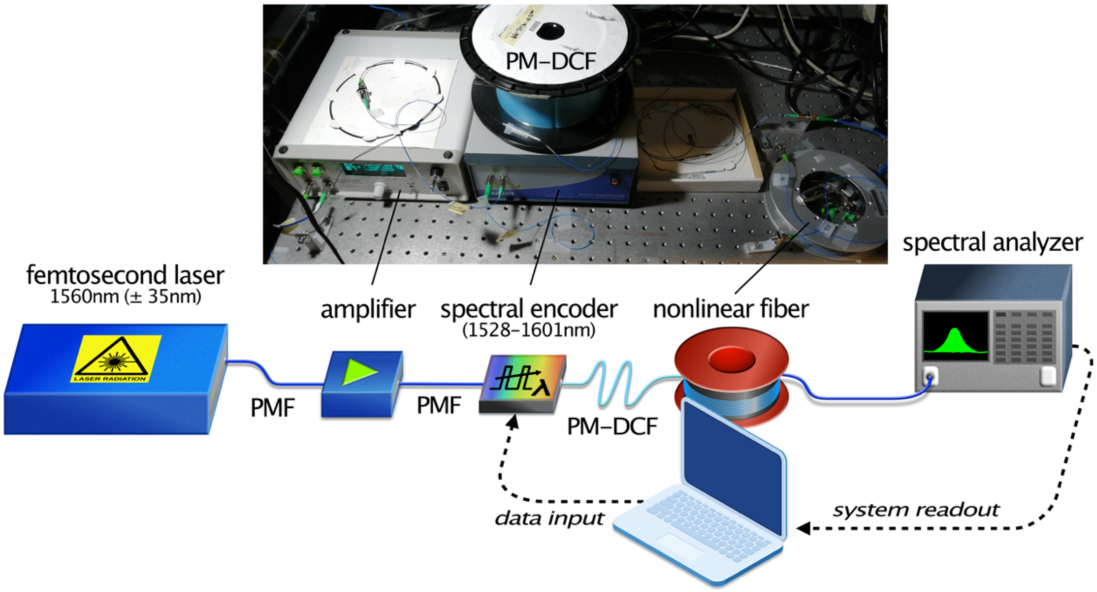

Fig. S1.

Schematic experimental setup of the implemented transient optical neural emulator based on high-order soliton fission. All incorporated fibers and fiber devices are polarization-maintaining. The inset depicts a photograph of the optical processing unit, showing the compact footprint of the system. PMF – polarization-maintaining fiber, PM-DCF – polarization-maintaining dispersion compensating fiber.

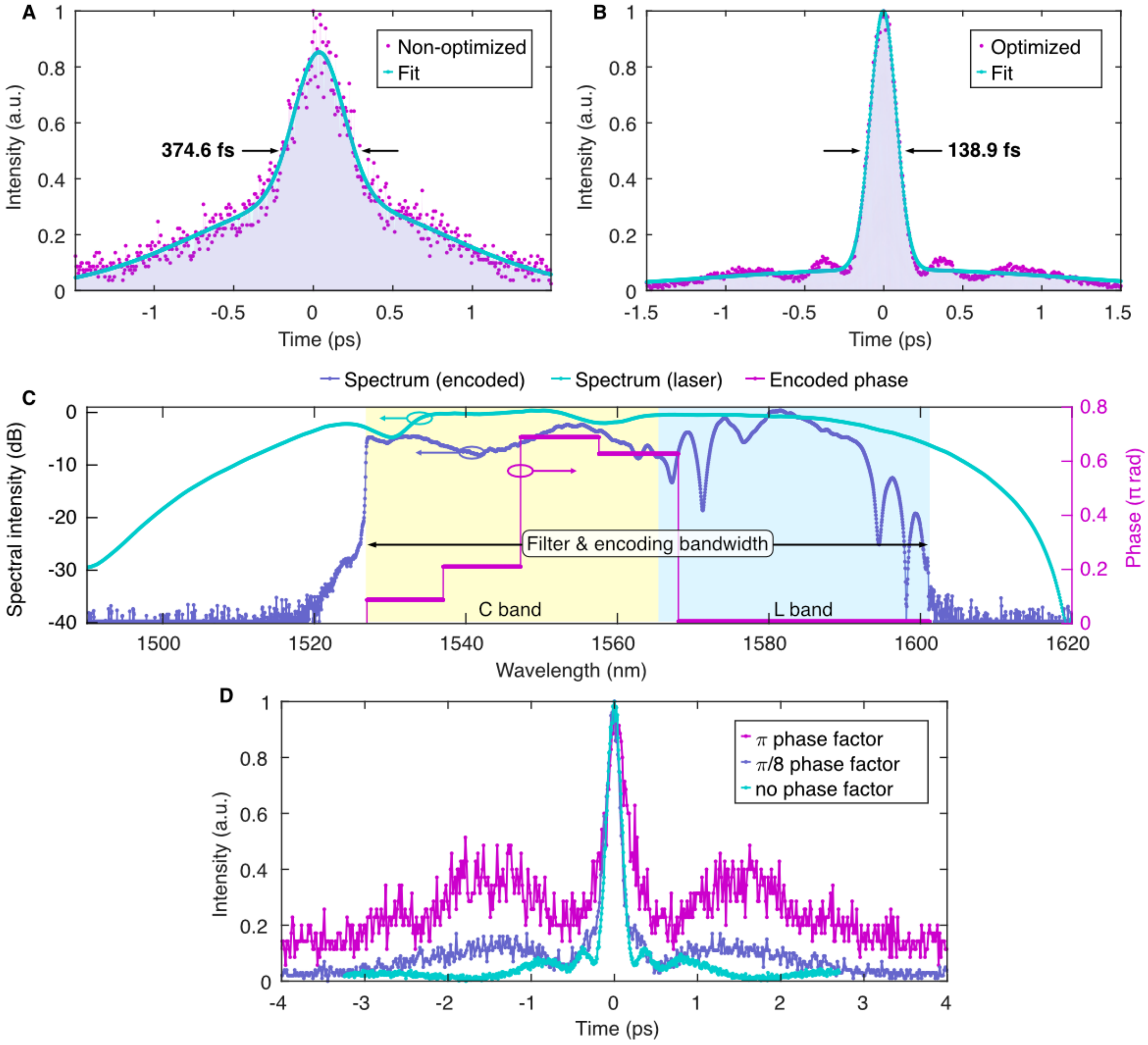

Fig. S2.

Spectro-temporal characterization of the information carrier pulse. (**A**) Autocorrelation trace before phase optimization measured in front of the nonlinear fiber. (**B**) Autocorrelation trace after phase optimization measured in front of the nonlinear fiber. In both cases, an autocorrelation correction factor of 0.707 was applied for the full width at a half maximum retrieval (i.e., pulse width labels in the figures). (**C**) Spectrum of the frequency comb pump laser (cyan), and spectrum after amplification and filtering through the programmable filter before entering the HNLF (purple). The C- and L-band covered by the programmable filter are highlighted in yellow and light blue, respectively. Here we show an example of a phase encoding mask (specifically for the case of an IRIS data set, in magenta) including four feature levels between 1528 nm and 1568 nm. (**D**) Autocorrelation traces after pulse optimization and no phase mask (cyan curve), a random phase mask of maximal magnitude π/8 (purple curve), and a random phase mask of maximal magnitude π (magenta curve).

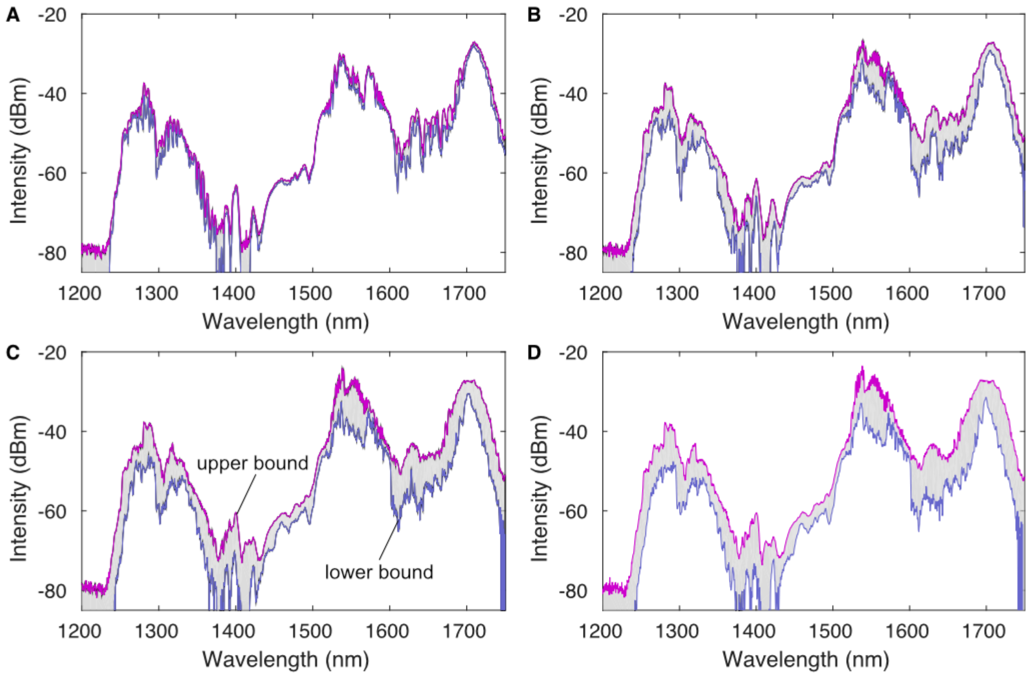

Fig. S3.

System sensitivity to the altitude and spectral location of a phase change in the pump spectrum. The panels show the measured output spectra of the HNLF for a continuous shift (from 1528 nm to 1600 nm) of a single bit (1 nm width) encoded in the spectral phase of the input pulse, with (**A**) 0.1 π phase shift, (**B**) 0.4 π phase shift, (**C**) 0.7 π phase shift, and (**D**) 1.0 π phase shift. The grey area in the plots indicates the individual spectra for all bit positions, and the colored spectra (blue and magenta) illustrate the minimum and maximum of each phase configuration (i.e., lower and upper bounds).

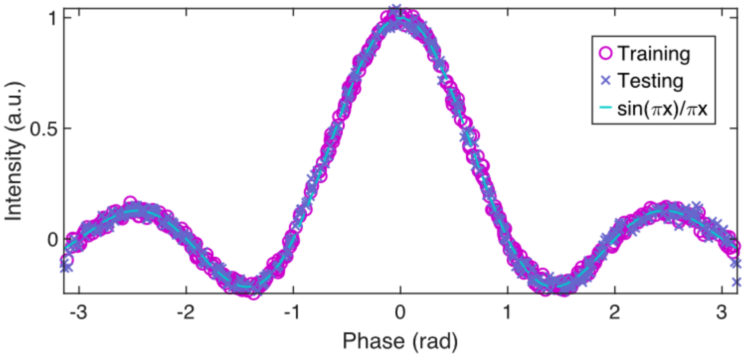

Fig. S4.

Results for the universal function regression of a *sinc* function. A significant low training error of 3.05e-4 (RMSE) and test error of 0.0175 (RMSE) indicate good function approximation capabilities of our experimental system.

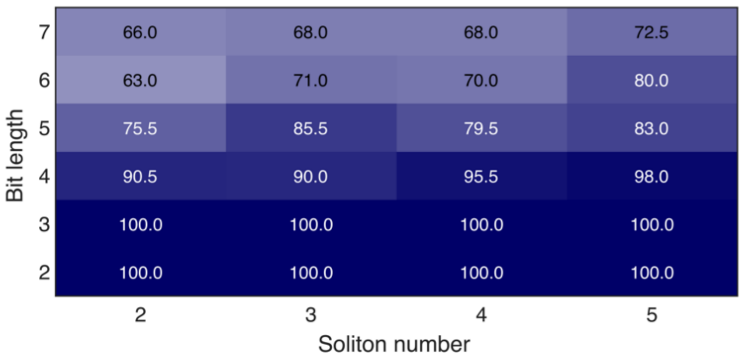

Fig. S5.

Training results (in %) for the n-bit parity task in the read-out configuration, where the number of bins is chosen to equal the bit length.

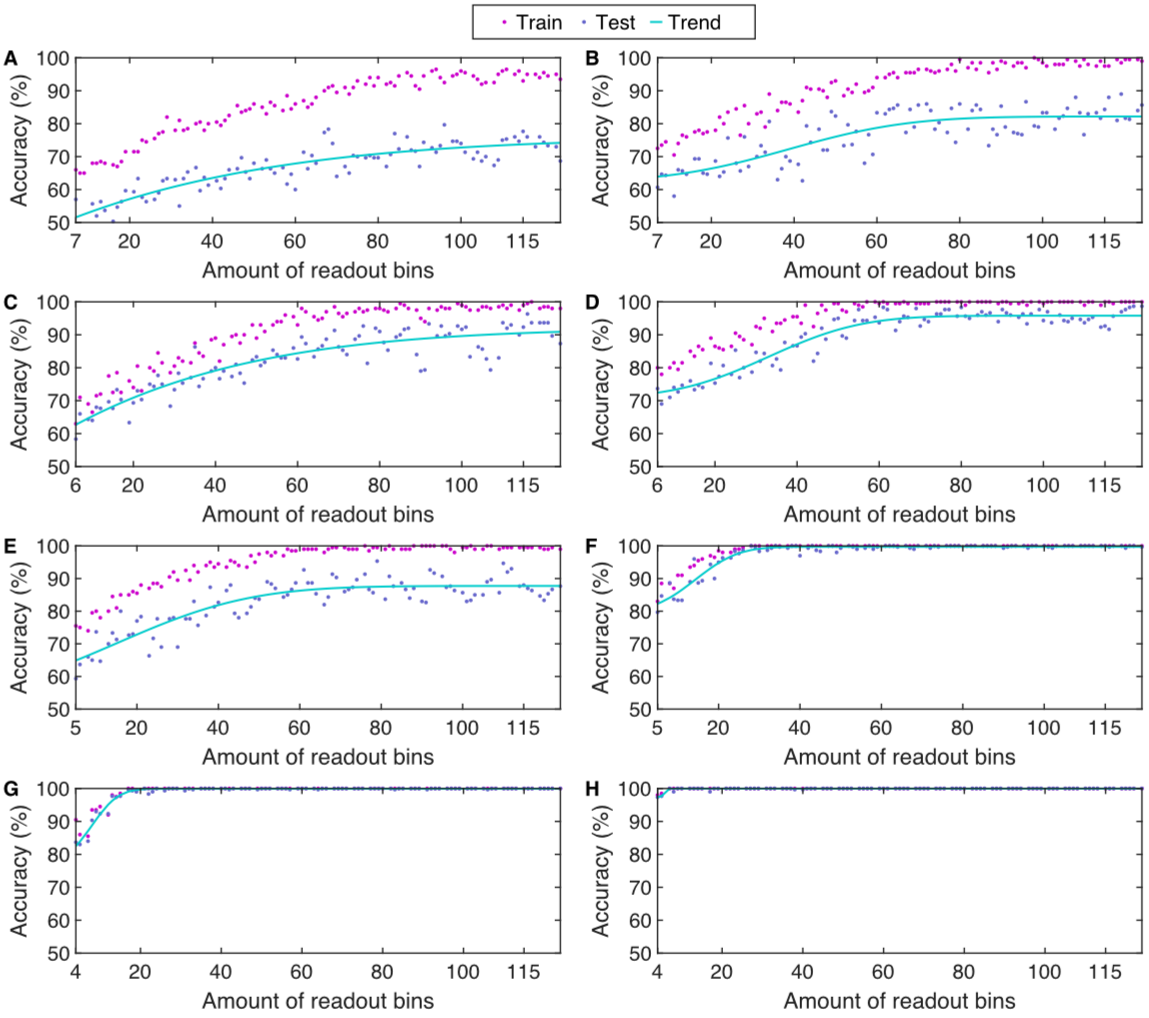

Fig. S6.

Experimental results for the n-bit parity test for different readout configurations. (**A, C, E, G**) Results for a soliton number of N=2 for (**G**) 4, (**E**) 5, (**C**) 6, and (**A**) 7 bit. (**B, D, F, H**) Results for a soliton number of N=5 for (**H**) 4, (**F**) 5, (**D**) 6, and (**B**) 7 bit.

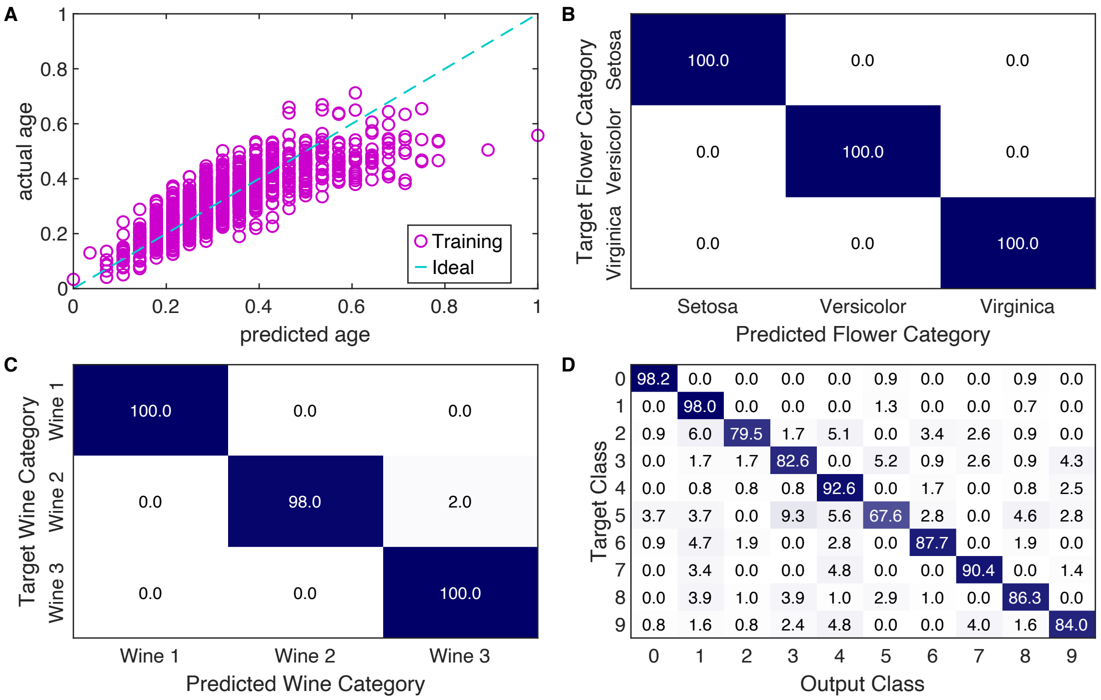

Fig. S7.

Training results for the different task-dependent benchmark tests. (**A**) Abalone age prediction (RMSE = 0.0684), (**B**) IRIS flower classification (accuracy 100%), (**C**) WINE classification (accuracy 99.3%, corresponding to a single misclassification), (**D**) MNIST handwritten digit recognition (accuracy 87.2%).

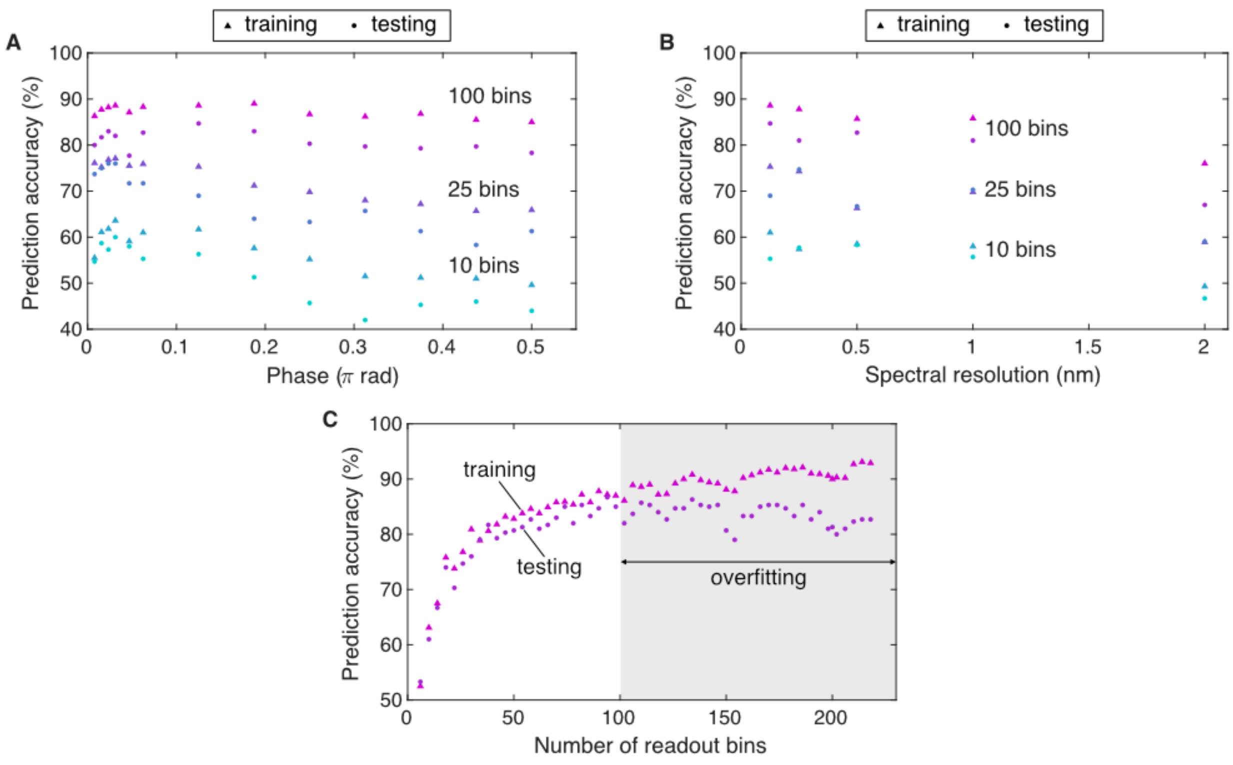

Fig. S8.

Dependence of the prediction accuracy of MNIST on phase altitude, spectral resolution, and number of readout bins. (**A**) Prediction accuracy over the encoding phase for various numbers of readout bins and 0.125 nm resolution. (**B**) Prediction accuracy vs. resolution of the spectral recordings for various numbers of readout bins and a π/8 encoding phase. (**C**) Prediction accuracy vs. the number of readout bins used to train the linear classifier for a π/8 encoding phase and 0.5 nm resolution.

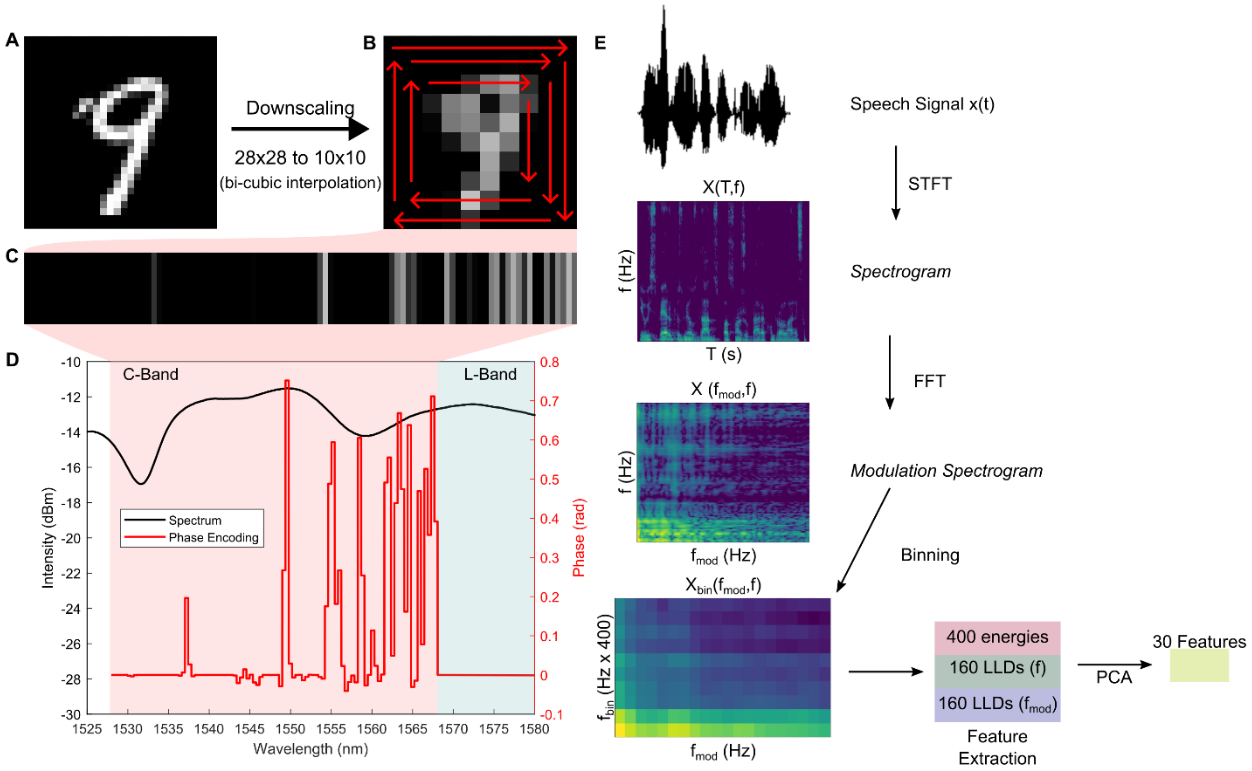

Fig. S9.

Illustration of the pre-processing for the MNIST and COVID-19 data sets. (**A** to **D**) Downscaling of the MNIST images (**A**) using a bicubic interpolation method. We employed (**B**) a spiral-unwrapping for (**C**) flattening the matrix (10×10) into a vector (1×100). The vector is encoded as (**D**) spectral phase offset on the input pulse in the optical C-band (1528 nm - 1567 nm) only. (**E**) Downscaling of the audio samples of the COVID-19 speech data. STFT – short time Fourier transform, FFT – fast Fourier transform, LLDs – low level descriptors, PCA – principal component analysis.

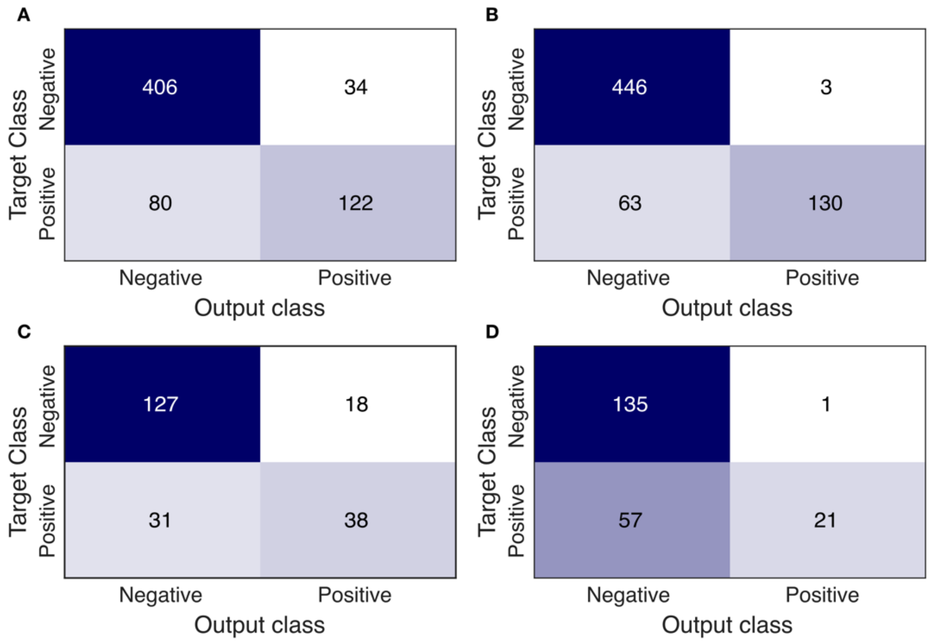

Fig. S10.

Results for the COVID-19 diagnosis from digital speech audio recordings. Training results obtained with (**A**) the experimental fiber-based ELM implementation (UAR = 0.7633), and (**B**) the digital SVM (UAR = 0.8334). Testing results obtained with (**C**) the experimental fiber-based ELM implementation (UAR = 0.7133), and (**D**) the digital SVM (UAR = 0.631).

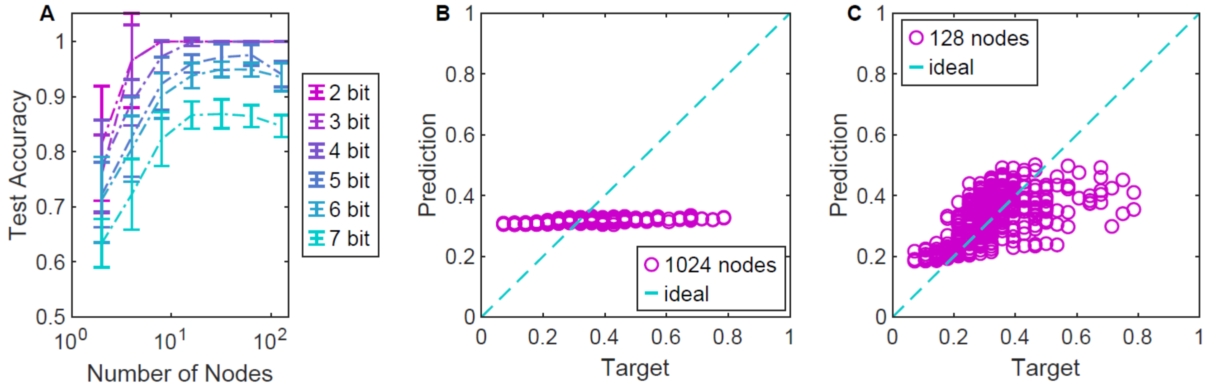

Fig. S11.

Digital neural network performance. (**A**) Test accuracy of the n-bit parity problem for digital neural networks with an increasing number of nodes (the x-axis only shows the number of nodes in the first layer). The error bars indicate the standard deviation over 50 different runs, the data points correspond to the average accuracy. (**B** and **C**) Comparison between (**B**) the mean square error (MSE) and (**C**) KL-divergence as a loss metric for the Abalone data set. Here we present the test samples of best performing neural networks after training: (**B**) a 2-layer network (1024 nodes in the first layer) which resulted in an RMSE of 0.118, and (**C**) a 2-layer network (128 nodes in the first layer) which resulted in an RMSE of 0.095.

Table S1.

Training hyperparameters for the different benchmark tasks shown in the present work. CV – cross-validations.

Dataset	**Training Samples**	**Test Samples**	**Training Ratio**	**Number of CVs**	**Input Features**	**Readout Bins**
Sinc	500	500	0.50	5	100	100
n-Bit Parity	200	300	0.40	5	varying	varying
IRIS	105	45	0.70	5	4	4
WINE	124	54	0.70	4	13	18
Abalone	1500	500	0.75	5	8	31
MNIST	1200	300	0.80	5	100	94
COVID-19	642	214	0.75	0	30	119

Table S2.

Comparison of different optical neuromorphic processing approaches. ELM – extreme learning machine, EOM – electro-optic modulator, MM – multi-mode, SM – single-mode, PD – photodiode.

**Platform**	**Task**	**Test Accuracy**	**Nonlinearity**	**Reference**
On-chip accelerator	IRIS	97.4%	PD ($I \propto E^2$)	*Ref.*[14]
On-chip accelerator	IRIS	96.67%	Digital function	*Ref.*[15]
EOM ELM	IRIS	93.9%	PD ($I \propto E^2$)	*Ref.*[16]
EOM ELM	WINE	97.5%	PD ($I \propto E^2$)	*Ref.*[16]
On-chip accelerator	WINE	97.22%	Digital function	*Ref.*[15]
EOM ELM	MNIST	90-92%	PD ($I \propto E^2$)	*Ref.*[17]
Free-space ELM	MNIST	92.2%	PD (saturation)	*Ref.*[18]
Free-space ELM	Abalone	0.12 (RMSE)	PD (saturation)	*Ref.*[18]
MM fiber	Abalone	0.126 (RMSE)	MM coupling	*Ref.*[19]
MM fiber	Sinc	0.0671 (RMSE)	MM coupling	*Ref.*[19]
SM fiber	Sinc	0.0175 (RMSE)	Soliton Dynamics	*Current work*
	Abalone	0.0686 (RMSE)		
	IRIS	100%		
	WINE	100%		
	MNIST	86.7%		

Table S3.

Overview of the achieved performance of the neural network primitives and their hyper-parameters. ϕ refers to the activation function applied to the output of a hidden layer. The column rate denotes the learning rate of the SGD optimizer. In the case of Abalone, the results are presented for the KL-divergence loss.

Task	L_H		***Hyper-parameters***		***Test Accuracy***
	**Number**	**Nodes**	ϕ	**rate**	
IRIS	1	4	tanh	.01	98.1%
WINE	1	256	tanh	.01	100%
Abalone	2	128/16	tanh	.0001	0.095 (RMSE)
MNIST	1	32	tanh	.005	88.4%

Table S4.

Overview of the artificial neural network (ANN) parameters and calculated FLOPs, as well as energy requirements for one inference step (input to hidden layer) of both TONE and competing digital ANN primitives, shown for the different tasks studied in the present work (see Fig. 3 in the main text).

**Task**	**Input Features**	**Hidden Nodes**	**FLOP** (input to hidden layer)	**Energy Consumption ANN Primitive**	**Energy Consumption TONE**
IRIS	4	4	20	1 nJ	0.089 nJ
WINE	13	8	112	5.6 nJ	
Abalone	8	128/16	3216	160.8 nJ	
MNIST	100	32	3232	161.6 nJ	
COVID-19	30	–	60	3 nJ	

Table S5.

Energy requirements of a single-pulse inference for different wave-based computing implementations.

Reference	Nonlinear mechanism	Energy per inference
This work	Soliton-fission	89 pJ (maximum used)
Ref. [19]	Nonlinear multi-mode coupling	320 nJ
Ref. [20]	Second-harmonic generation (on-chip)	50 pJ
Ref. [9]	Second-harmonic generation (free-space)	>nJ
